# Mechanistic Insights for Plasma-Catalytic CO_2_ Reduction over TiO_2_ in a Dielectric Barrier Discharge Reactor

**DOI:** 10.1021/acsengineeringau.5c00092

**Published:** 2026-01-27

**Authors:** Diego Alexander Gonzalez-Casamachin, Yuyang Hu, Srinivas Rangarajan, Jonas Baltrusaitis

**Affiliations:** Department of Chemical and Biomolecular Engineering, 1687 Lehigh University, Bethlehem, Pennsylvania 18015, United States

**Keywords:** plasma, TiO_2_, catalyst, kinetics, CO_2_, dissociation

## Abstract

Reaction kinetics experiments coupled with phenomenological kinetic modeling and parameter estimation are used to elicit insights into the mechanism and active sites for the plasma-catalytic dissociation of CO_2_ on TiO_2_. Experimental and model insights showed that gas-phase reactions contribute at least two-thirds of the overall product formation at explored conditions; weak temperature dependence, strong sensitivity to specific energy input (SEI), apparent first order in CO_2_, and positive influence of cofed argon (Ar) and oxygen (O_2_) for the gas-phase contributions all suggest that expected plasma reaction steps such as electron-impact and high-energy collisions are the dominant modes for CO_2_ dissociation. The Arrhenius-like expression for gas contributions resulted in a preexponential of 4.40 × 10^–3^ s^–1^, an *E*_SEI,g_ of 7.90 × 10^–4^ mol/kJ, and an *E*_a,g_ of 1.00 × 10^–3^ J/mol. For surface contributions, the small apparent barrier of 16.3 kJ/mol, relatively weaker dependence on SEI, first-order dependence on CO_2_, and insensitivity to cofed Ar and O_2_ all point to CO_2_ dissociation on TiO_2_ surface facets without vacancies and aided by plasma (leading to vibrationally excited CO_2_ and/or a reactive surface with significant surface charge accumulation). The Arrhenius-like expression resulted in a preexponential of 7.81 × 10^–2^ s^–1^, an *E*_SEI,s_ of 1.90 × 10^–3^ mol/kJ, and an *E*_a,s_ of 1.63 × 10^4^ J/mol. The derived kinetic model further enabled a systematic evaluation of the effect of inputs (plasma power, flow rate, CO_2_ inlet concentration, and temperature) to identify process trends and optimal operating conditions.

## Graphical abstract

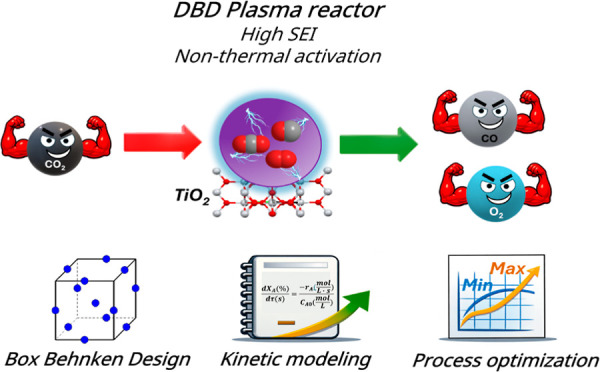

## 1 Introduction

The increasing concentration of carbon dioxide (CO_2_) in the atmosphere has become a critical environmental concern due to its significant role in global warming and climate change.^1^ To mitigate the impact of rising CO_2_ emissions, various strategies have been developed, including carbon capture and storage (CCS), carbon utilization technologies, and direct conversion of CO_2_ into valuable fuels and chemicals. Among these approaches, plasma-assisted CO_2_ conversion has emerged as a promising alternative due to its ability to operate under atmospheric conditions and relatively low temperatures while enabling the efficient dissociation of CO_2_ molecules.^2^

Nonthermal plasma (NTP) technology has been extensively explored for CO_2_ reduction, particularly in dielectric barrier discharge (DBD) and packed bed plasma.^3–6^ Dielectric barrier discharge (DBD), as a type of nonequilibrium plasma, is an excellent source of plasma containing energetic electrons with electron energies of 1–10 eV that can be generated at ambient temperature under atmospheric pressure.^7^ This is the ideal energy range for the excitation of atomic and molecular species capable of breaking the strong C=O bonds in CO_2_, facilitating its conversion into CO, O_2_, and other valuable products.^8–10^ Nevertheless, a key challenge in plasma-based CO_2_ conversion is the low selectivity for CO and the limited energy efficiency of plasma-only processes, leading to significant energy losses and restricted product yields.^11^

To address these challenges, researchers have introduced catalysts and dielectric packing materials into plasma reactors, which enhance conversion efficiency by optimizing electron interactions and promoting surface reactions.^12,13^ Plasma catalysis has, in particular, garnered increasing attention due to its potential to improve both conversion rates and energy efficiency.^14^

Several studies have explored CO_2_ dissociation in packed-bed DBD reactors using metal catalysts such as Ni, Mg, and Pd, coated on Al_2_O_3_ supports, as well as nonmetallic materials like quartz wool and zeolite.^15,16^ In this context, oxides such as TiO_2_ have also been employed to enhance CO_2_ dissociation.^8^ Mei et al. evaluated the DBD plasma conversion of pure CO_2_ at low temperatures using a TiO_2_ photocatalyst^8^ and showed that the combinations resulted in a substantial increase in CO_2_ conversion and energy efficiency (by a factor of 1.6). Ray et al. employed TiO_2_ mixed with g-C_3_N_4_, which resulted in a CO_2_ conversion of 7.5% at a specific energy input (SEI) of 4.6 J/mL.^17^

TiO_2_ is a well-known semiconductor with advantages including chemical stability, simple preparation, environmental protection, and low cost.^18–20^ TiO_2_ has also been employed as a packing material in DBD plasma reactors to enhance the reduced electric field, which can increase the mean electron density. For instance, Tu et al. investigated the influence of TiO_2_ on the physical characteristics of nitrogen DBD using a combination of electrical and optical emission spectroscopy. Their results demonstrated that the presence of TiO_2_ significantly increases the vibrational temperature of N_2_ in the DBD, suggesting that plasma–catalyst interactions strongly influence the electron energy distribution function. Specifically, TiO_2_ enhances the electron density in the high-energy tail of the distribution function.^21^ Finally, studies have shown that the presence of diluent gases such as Ar, N_2_, or O_2_ in CO_2_ gas mixtures, along with the use of catalysts, significantly influences CO_2_ decomposition in packed-bed plasma reactors at low temperatures and ambient pressure.^22–24^

Mei et al. attributed the observed enhancement in TiO_2_ to the combined effects of plasma-induced electron excitation and the catalytic properties of photocatalysts, which induce plasma-physical effects such as the enhancement of the electric field and the generation of more energetic electrons and reactive species.^8^ Mechanisms of photocatalytic CO_2_ dissociation have also been proposed, viz., the creation of oxygen vacancies where CO_2_ can dissociate in a facile manner. However, a detailed understanding of the mechanism and active sites of this dissociation chemistry on TiO_2_ is lacking. In this study, we address this critical gap by conducting a comprehensive reaction kinetics study, coupled with kinetic modeling of the dissociation of CO_2_ in a DBD plasma reactor using TiO_2_ as a catalyst. We employ the model we learned to understand the plausible active site for CO_2_ dissociation on TiO_2_ and identify optimal conditions for energy-efficient CO_2_ utilization.

## 2 Experimental Section

### 2.1 Titanium (IV) Oxide Catalyst

Titanium (IV) oxide (TiO_2_) was purchased from Sigma-Aldrich and used as received. According to the supplier specifications, the material had an average particle size of 21 nm and a specific surface area of 35–65 m^2^ g^–1^.

### 2.2 Experimental Setup and Catalytic Performance

CO_2_ decomposition was carried out in a DBD plasma reactor, as illustrated in Figure 1. In this setup, 200 mg of commercial TiO_2_ was placed in the discharge zone of the reactor, dispersed uniformly on 0.5 g of quartz wool with a bulk density of 2.2 g/cm^3^. The reactor featured a stainless-steel inner electrode with a 16 mm diameter and a glass tube with a 20 mm inner diameter. Copper tape wrapped around the glass tube functioned as the outer electrode, and the tube had a total length of 140 mm. To raise the temperature to 140 °C, an electric heater was positioned around the outer electrode. An SRI 8610C gas chromatograph using argon as a carrier gas was employed to measure the gas products. The GC device was equipped with a thermal conductivity detector (TCD), and gaseous products such as CO_2_ and CO were separated using a combination of MS5A and A Hayesep D column along with a TCD. The reaction temperature was measured by a K-type thermocouple placed near the reactor outlet. CO_2_ quantification, CO_2_ conversion, SEI (kJ/mol) energy intensity, energy efficiency (EE, total and nonthermal), and carbon balance (CB) were evaluated via (eq 1) through (eq 6).

1 $$C_{{CO}_{2}}^{in/out}\;\left(\frac{scc}{\min}\right)=\left(\left(GC\;area\;{CO}_{2}^{in/out}\right)\times \alpha \times \beta \right)\times F_{total}$$

2 $$X_{{CO}_{2}}\;\left(\%\right)=\left(\frac{C_{{CO}_{2}}^{in}-C_{{CO}_{2}}^{out}}{C_{{CO}_{2}}^{in}}\right)\times 100$$

3 $$SEI\;\left(\frac{kJ}{mol}\right)=\frac{discharge\;power\;\left(kW\right)}{total\;gas\;flow\;rate\;\left(\frac{L}{s}\right)}\times 22.4\;\left(\frac{L}{mol}\right)$$

4 $$energy\;intensity\;\left(\frac{kJ}{mmol}\right)=\frac{plasma\;power\;\left(kW\right)+heating\;power\;\left(kW\right)}{{CO}_{2}\;converted\;\left(\frac{mmol}{s}\right)}$$

5 $$EE_{total}\;\left(\%\right)=\frac{heat\;of\;CO\;combustion\;\left(\frac{kJ}{mmol}\right)}{energy\;intensity\;\left(\frac{kJ}{mmol}\right)}\times 100$$

5b $$EE_{plasma}\;\left(\%\right)=\frac{heat\;of\;CO\;combustion\;\left(\frac{kJ}{mmol}\right)}{plasma\;power\;\left(kW\right)}\times {CO}_{2}\;converted\;\left(\frac{mmol}{s}\right)\times 100$$

6 $$CB\;\left(\%\right)=\frac{C_{{CO}_{2}}^{out}+C_{CO}^{out}}{C_{{CO}_{2}}^{in}}\times 100$$

where $C_{{CO}_{2}}^{in}$ (scc/min) represents the inlet flow of the CO_2_, $C_{{CO}_{2}}^{out}$ (scc/min) corresponds to the outlet CO_2_ flow, *C*_CO_^out^ (scc/min) denotes the outlet CO flow rate, and *F*_total_ denotes the total flow rate. The outlet concentrations of CO_2_ and CO were determined using gas chromatography (GC) based on calibration curves. The GC area for species *i* corresponds to the inlet or outlet signal of that compound, and the calibration constants α and β were obtained from a linear calibration curve for each species, as described in a previous study.^12^ For the calculation of energy efficiency, the standard enthalpy of CO combustion was used with ΔH_298K_^°^ = 283.3 kJ/mol. While the heating power for the lab reactor is supplied by electricity, the heating requirements of an industrial reactor will be met through nonelectrical sources. Further, industrial reactors can benefit from heat integration with other utilities and streams in the plant, and the rate of heat loss from these reactors is much less (compared to lab-scale). Therefore, from a scale-up standpoint, efficiency without taking into account heating power is useful; we define this as the plasma energy efficiency (EE_plasma_).

**Figure 1:**
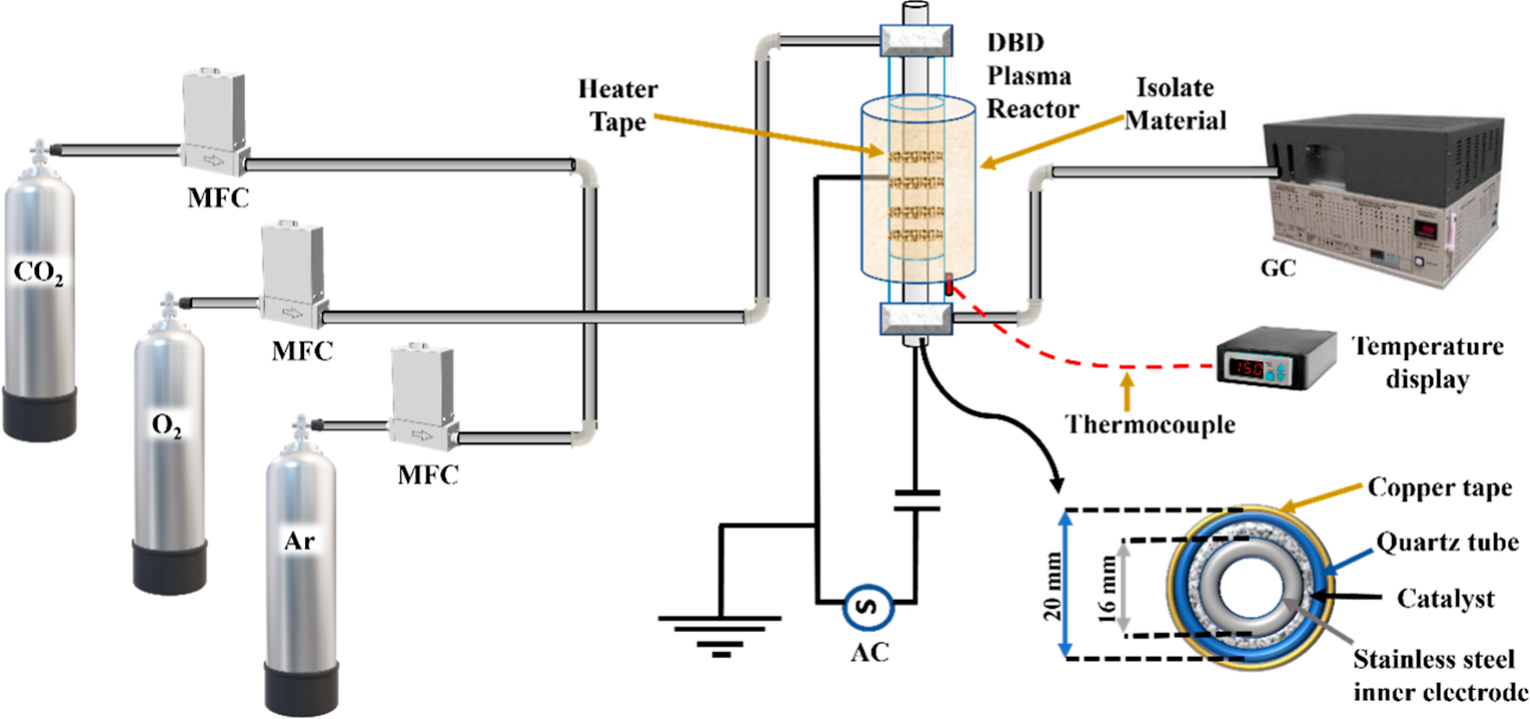
Schematic diagram of the experimental setup.

### 2.3 Design of Experiments

A four-factor, three-level Box–Behnken design (BBD) was implemented to evaluate the individual effects and interactions influencing the synergistic catalysis of CO decomposition using DBD plasma and TiO_2_ catalysts as well as to optimize the reforming conditions. Preliminary experiments were conducted to establish suitable boundary conditions for the BBD (Table SI1). The plasma-catalytic decomposition of CO_2_ was carried out under ambient conditions. The four independent process parameters included discharge power (*P*_1_), temperature (*P*_2_), CO_2_ concentration (*P*_3_), and flow rate (*P*_4_). CO_2_ (*Y*) was considered as a response factor. Table 1 presents the three levels for each parameter, while the experimental design matrix is summarized in Table SI1 from runs 65 to 91. In total, the design generated 27 experimental runs. All conditions were run with and without TiO_2_ to distinguish the gas-phase and surface contributions, thus, for a total of 54 experiments. Additional experiments (117) from a partial higher-level BBD were employed (mostly data with a catalyst) to fit the kinetic model. Table SI1 presents the results obtained, comprising 171 runs with a catalyst and 43 runs without a catalyst, for a total of 214 experiments. Conversion of CO_2_ was low in most cases (∼15% or less), and ∼34 runs had a conversion >20%.

**Table 1:** Process Parameters for the Box–Behnken Design

process parameters	unit	–1	0	1
discharge power (*P*_1_)	Watts	35	42.5	50
temperature (*P*_2_)	°C	140	155	170
CO_2_ concentration (*P*_3_)	%	94	97	100
flow rate (*P*_4_)	mL/min	70	77.5	85

All experiments used to evaluate the catalyst effect were conducted in triplicate, and for each experiment, the outlet gas composition was sampled three times to reduce the systematic and analytical uncertainty. For the Box–Behnken study, each experimental condition was sampled three times, while replication of operating conditions was inherently provided by the design of experiments framework. The reliability of the main experimental data was confirmed through carbon balance analysis, as presented in Tables SI2–SI5.

### 2.4 Kinetic Analysis

#### 2.4.1 Mathematical Model

A packed bed reactor (PBR) model (eq 7) with simplified (phenomenological) kinetics was developed to describe the gas-phase kinetics and surface kinetics (eqs 8 and 9). In particular, the conversion *X*_A_ (unitless or %) of CO_2_ in a PBR with residence time τ (in sec) is given by

7 $$\frac{d\left(X_{A}\right)}{d\tau }=k_{g}{C_{A0}}^{m-1}{\left(1-X_{A}\right)}^{m}+k_{s}{C_{A0}}^{n-1}{\left(1-X_{A}\right)}^{n}$$

8 $$k_{g}=A_{g}e^{-E_{a,g}/RT}f_{g}\;\left(SEI,C_{Ar}\right)$$

9 $$k_{s}={A_{s}e}^{-E_{a,s}/RT}f_{s}\;\left(SEI,C_{Ar}\right)$$

where *k*_g_,*A*_g_,*A*_s_ and *k*_s_,*A*_s_,*A*_g_ are the rate constants (in $\frac{L^{m-1}}{{mol}^{m-1}\cdot s}$ ), pre-exponential factor (in $\frac{L^{m-1}}{{mol}^{m-1}\cdot s}$ ), and activation barrier (based on the Arrhenius formulation, in J/mol) for the gas and surface reactions (*k*_s_ and *A*_s_ in $\frac{L^{n-1}}{{mol}^{n-1}\cdot s}$ ), respectively; *m* and *n* are the reaction orders of gas and surface reactions, respectively; functions *f*_s_ and *f*_g_ are generic expressions that capture the SEI and the concentration of argon cofeed (*C*_Ar_). Their detailed explanations are given in Section 1.1 of Supporting Information. The values of these parameters are shown in Table 2. The model in eq 7 effectively assumes that the CO_2_ dissociation is irreversible (i.e., the reverse reaction is neglected); this is reasonable since the goal is to obtain kinetic parameters from low-conversion data.

**Table 2:** Best Fit Values of the Kinetic Model

	*A*_g_ (s^–1^)	*E*_SEI,g_ (mol/kJ)	*E*_a,g_ (J/mol)	β_g_	*m*
value	4.40 × 10^–3^	7.90 × 10^–4^	1.00 × 10^–3^	4.5	1
standard deviation	0.0549	2.034 × 10^–4^	2.230 × 10^4^	2.336	1.193

	*A*_s_ (s^–1^)	*E*_SEI,s_ (mol/kJ)	*E*_a,s_ (J/mol)	β_g_	*n*
value	7.81 × 10^–2^	1.90 × 10^–3^	1.63 × 10^4^	-	1
standard deviation	0.0027	9.038 × 10^–5^	49.729	-	0.0033

The parameters of the model are fit to experimental data by minimizing an objective function that minimizes model-error mismatch and evaluates the performance of the model using the mean relative error (MRE) and adjusted *R* ^2^, as described in Table 3.

**Table 3:** Summary of Model Performance

	MRE_gas_	MRE_total_	adjusted *R*_gas_^2^	adjusted *R*_total_^2^
value	22.00%	16.66%	0.8850	0.7965

#### 2.4.2 Model Evaluation

The fitted model was used to analyze the effect of changes in the flow rate, inlet concentrations, temperature, and plasma power. The scope of these 4 operation variables can be seen in Table SI6. To this end, the following metrics were used: (1) total rate of conversion of CO_2_ (*R*_CO_2__)) as given in eq 10, (b) energy intensity (EI) as given in eq 4, (c) energy efficiency (EE_total_) as given in eq 5, and (d) contribution of the gas phase (ζ) to the overall conversion, as described in eq 11.

10 $$R_{CO_{2}}\left(\frac{mmol}{h}\right)=F_{total}\left(\frac{mL}{h}\right)\times X_{{CO}_{2}}\times C_{CO_{2}}^{in}\;\left(\frac{mmol}{mL}\right)$$

11 $$Gas\;contribution\;\left(\zeta \right)=\frac{X_{CO_{2},nocat}}{X_{CO_{2},w/cat}}$$

## 3 Results and Discussion

### 3.1 Effect of Inlet CO_2_ Concentration

The concentration of CO_2_ was varied by diluting it with argon. While typically, such experiments are employed to obtain reaction order information, in plasma catalysis, it is known that diluent inert gases have a promotional effect by, e.g., increasing the electron density in the plasma.^22^ In particular, Xu et al.^25^ showed that (1) the concentration of high-energy electrons increases in inert conditions because of a reduction in the breakdown voltage (i.e., the minimum voltage to initiate a plasma discharge), thereby increasing the rate of electron impact reactions involving CO_2_ and (2) high-energy Ar atoms or Ar^+^ ions can form that can impact with CO_2_ to either promote them to high-energy states (or CO_2_^+^ ion) or dissociate the C–O bond, all of which can facilitate CO_2_ conversion in the gas phase. As shown in Figure 2, the level of CO_2_ conversion increases almost linearly with the concentration of the diluent gas, suggesting that introducing a carrier gas enhances the plasma-driven conversion. This is consistent with findings from plasma systems without packing materials, such as dielectric barrier discharge (DBD) reactors.^26^ The inclusion of the TiO_2_ catalyst in general increases the CO_2_ conversion. The catalytic contributions are determined to be the difference between the run with the catalyst and that without. Considering the low TiO_2_ loading, we expect that its introduction will not significantly change the physical characteristics of the plasma; therefore, the gas-phase contributions in the presence of the catalyst are nearly equal to the rate in the absence of the catalyst. We note that the catalytic contribution drops slightly with argon cofeed, indicating that the surface rate depends on the concentration of CO_2_.

**Figure 2:**
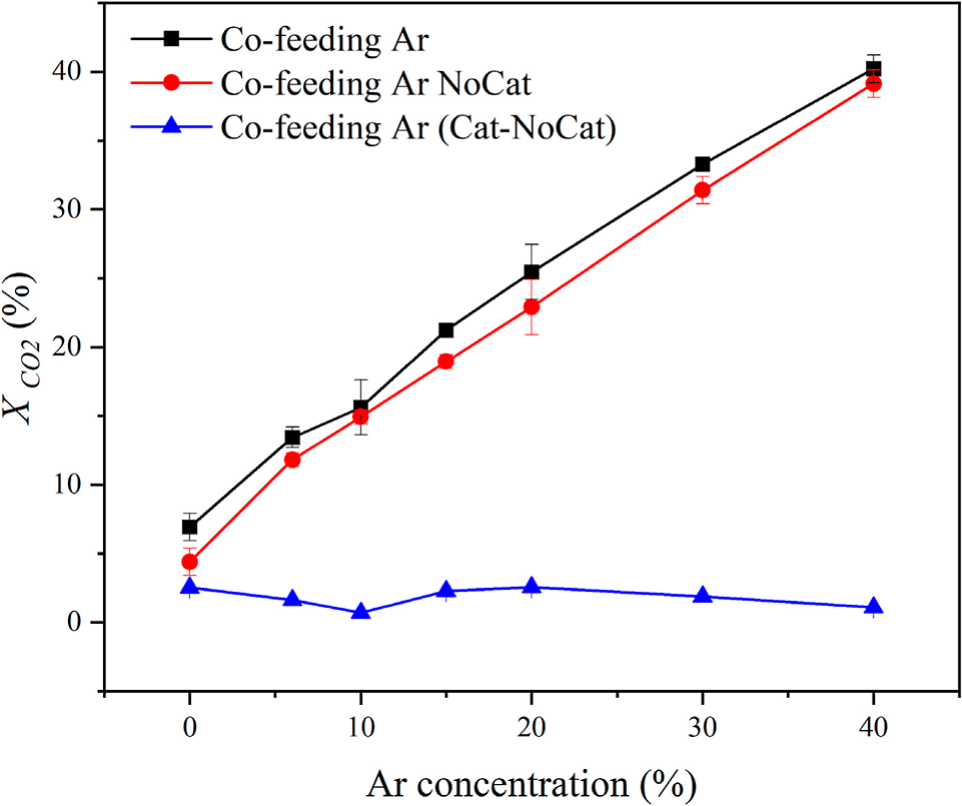
CO_2_ conversion versus concentration of argon. All other input factors are fixed. The flow rate is 70 ccm, the discharge power is 35 W, and the temperature is 140 °C. “Cat” and “NoCat” refer to runs with and without the catalyst, respectively. “Cat-NoCat” refers to the difference computed between these two sets of runs for the same experimental conditions. The lines are included to help in visualizing the trends.

### 3.2 Effect of the Specific Energy Input on CO_2_ Dissociation

Figure 3 illustrates the effect of specific energy input (SEI, kJ/mol) on the conversion of CO_2_ (*X*_CO2_) under plasma-assisted conditions, comparing runs with and without the TiO_2_ catalyst. Conversion increases with SEI, consistent with prior observations.^27^ The low loading of solid TiO_2_ ensures that the additional volume occupied by the catalyst is only 0.3% of the quartz wool volume; therefore, the gas-phase residence time remains unaffected. A higher SEI (either through increasing plasma power or decreasing flow rate) plausibly leads to increased microdischarge in the reactor, which in turn leads to a higher concentration of reactive species such as CO_2_ in electronically and vibrationally excited states, thereby promoting more effective CO_2_ dissociation.^8,25^

**Figure 3:**
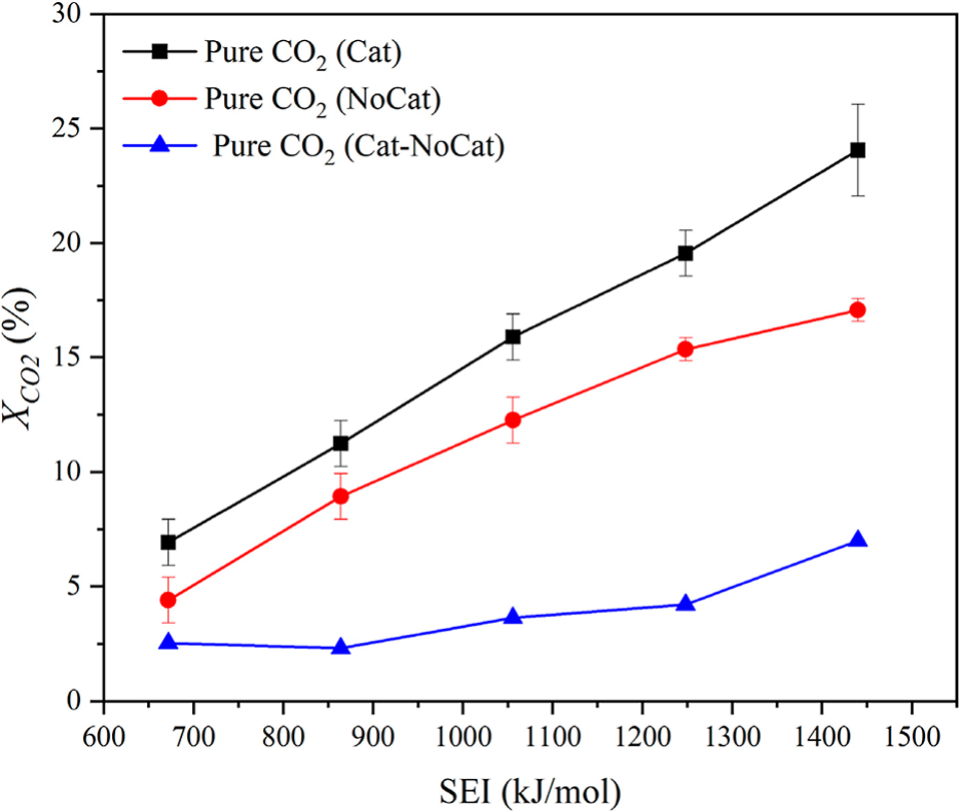
CO_2_ conversion versus SEI at a constant flow rate of 70 ccm, 140 °C, and pure CO_2_ feed. “Cat” and “NoCat” refer to runs with and without a catalyst, respectively. “Cat-NoCat” refers to the difference computed between these two sets of runs for the same experimental conditions. The lines are included to help in visualizing the trends.

### 3.3 Effect of the Temperature on CO_2_ Reduction

Figure 4 illustrates the effect of reactor temperature on CO_2_ conversion under steady-state plasma operation, both in the presence and absence of catalyst, over the temperature range of 140–170 °C. Previous studies have shown that CO_2_ dielectric barrier discharges operated at comparable specific energy densities reach a stabilized gas temperature of approximately 138 °C in the absence of a catalyst.^8^ The introduction of dielectric materials such as TiO_2_ has been reported to cause a slight increase in the steady-state temperature, typically to about 144–149 °C, which has been attributed to changes in discharge characteristics and enhanced inelastic electron–molecule interactions.^8^

**Figure 4:**
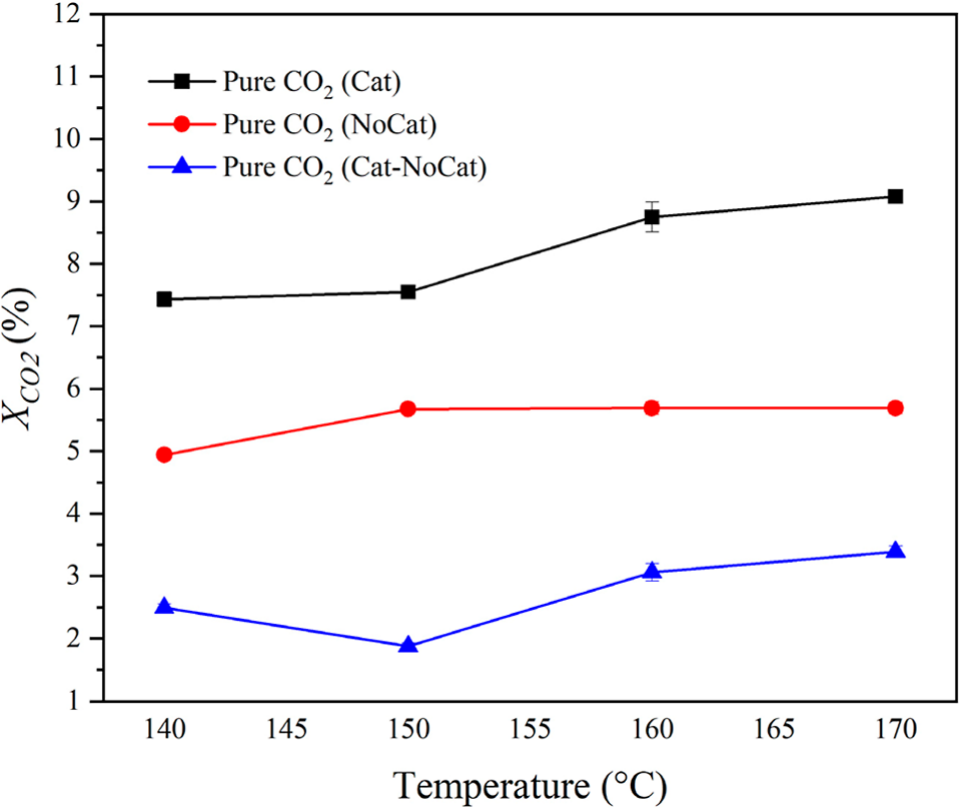
CO_2_ conversion versus temperature at a constant flow rate of 70 ccm, a plasma discharge power of 35 W, and pure CO_2_ feed. “Cat” and “NoCat” refer to runs with and without a catalyst, respectively. “Cat-NoCat” refers to the difference computed between these two sets of runs for the same experimental conditions. The lines are included to help in visualizing the trends.

Within this temperature regime, CO_2_ conversion increases with temperature for both catalytic and noncatalytic systems, consistent with enhanced plasma-driven reaction kinetics.^28–30^ Although a higher overall conversion is observed in the presence of TiO_2_, inferred from the difference between experiments with and without a catalyst (“Cat-NoCat” in the figure), the effect shows a weak dependence on temperature across the investigated range. This behavior indicates that temperature alone does not govern the catalytic effect. Rather, the results support the conclusion that gas-phase plasma reactions dominate CO_2_ conversion under the conditions examined.

### 3.4 Kinetic Modeling

The experimental data collected in this study were employed to fit the model shown in eqs 7 and 9. This first requires determining the functional forms of *f*_g/s_. Experimental results show a dependence of SEI on both gas-phase and surface reactions, while temperature only affects the surface reactions. Previous studies have shown that, akin to an Arrhenius dependence of kinetics on temperature, ln­(*k*) is proportional to the inverse of plasma power, or SEI.^31^ However, our data also show a superlinear relationship with SEI that can be captured with a scaled exponential term. The effect of Ar on the gas-phase kinetics also has a superlinear relationship, and we, therefore, again included a scaled exponential dependence; the effect of Ar on the surface kinetics is negligible, so we neglect this term. Accordingly, we assume

12 $$f_{g}\;\left(SEI,C_{Ar}\right)=e^{SEI\times E_{SEI,g}}e^{\beta C_{Ar}}$$

13 $$f_{s}\;\left(SEI,C_{Ar}\right)=e^{SEI\times E_{SEI,s}}$$

where *E*_SEI,g_ and *E*_SEI,s_ are coefficients akin to the apparent activation barrier and have units of mol/kJ and β is a constant to describe the contribution of inert gas Ar to the reaction, while *C*_Ar_ is the percentage of Ar in the feed gas.

Following the procedure outlined in Supporting Information (Section 1.2), the best regression results where the objective function is the smallest among all the independent optimization runs and the corresponding statistical metrics are shown in Tables 2 and 3, respectively. The results using a variation of eq 11, where an Arrhenius-like behavior is observed for SEI ( $e^{-E_{SEI}/SEI}$ ), are shown in the Supporting Information (Tables SI7 and SI8).

The pre-exponential factor of the surface is almost 17 times higher than the gas-phase reaction, while *E*_SEI,s_–2.4*E*_SEI,g_. As expected, the activation barrier for the gas-phase contribution is nearly zero, while the surface barrier is about 16.3 kJ/mol. The gas phase β, capturing the Ar dependence, is 4.5; therefore, ∼1% of Ar cofeed increases the kinetics by about 4.5%. A parity plot of the model vs experimental conversion is shown in Figure 5; clearly, the model is able to capture the data without bias (no consistent over/underprediction observed), and the mean relative error on combined (gas and surface) contributions is ∼16.71%. The performance of the model on runs without a catalyst has a mean relative error of 22.35% and an adjusted *R* ^2^ value of 0.87.

**Figure 5:**
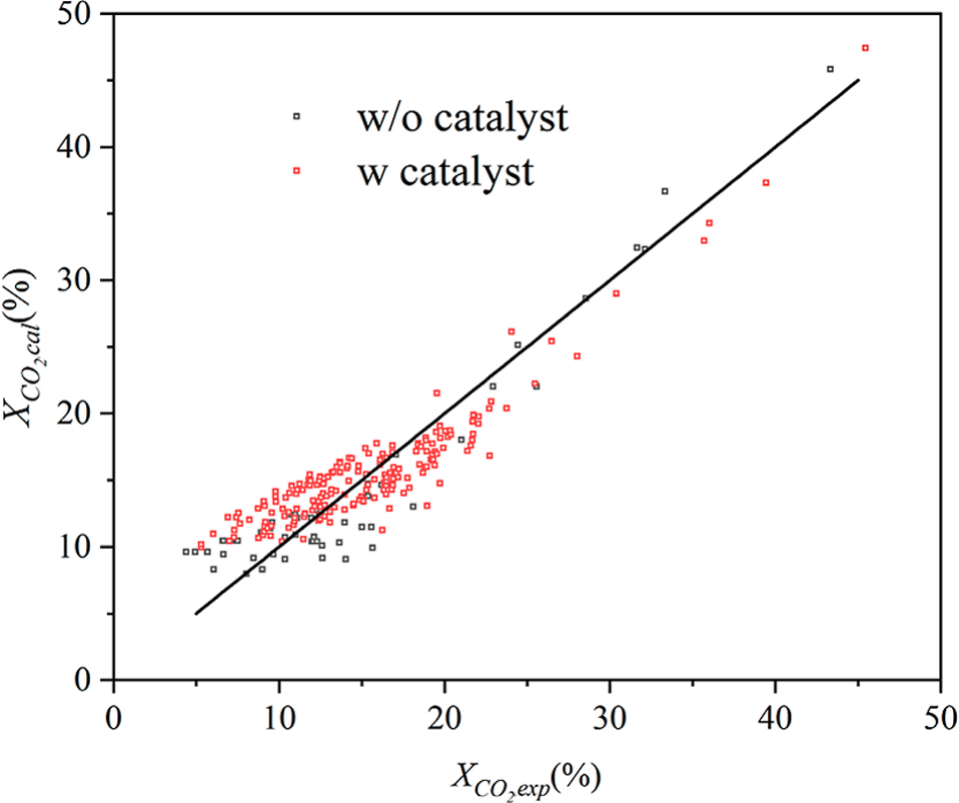
Parity plot of model vs experimental conversion with/without a catalyst. The black line represents the parity (*y* = *x*) line. Points above/below this line indicate over/underprediction.

### 3.5 Inference of the Reaction Mechanism

Interpretation of the kinetic modeling results can provide insights into the mechanism of plasma catalytic CO_2_ dissociation. In particular, the gas-phase constant ***k***_**g**_ is dependent on SEI but not on temperature; additionally, the model predicts a first-order dependence of gas-phase kinetics on CO_2_. These observations are consistent with arguments in prior experimental and computational literature that gas-phase dissociation of CO_2_ in nonthermal plasma is primarily through electron-impact reactions involving the reactant at the range of SEI values considered in this study.^28,32^ **β** = 4.5 for the gas-phase reaction is also consistent with the earlier discussion about the dependence of the CO_2_ dissociation kinetics on the inert gas. At 170 °C, SEI = 1000 kJ/mol, and no Ar cofeed, the model parameters indicate that gas-phase reactions contribute ∼77% of the net kinetics. The pre-exponential factor for the surface is higher than that of the gas-phase kinetics; however, the kinetics is dependent both on the SEI and temperature (with a larger activation barrier of 16.3 kJ/mol compared to the gas phase). Remarkably, the model predicts the order *n* = 1, i.e., first-order dependence on CO_2_, indicating that C–O scission is rate-determining.

Previous studies have postulated that the impact of electrons on the TiO_2_ surface, in the presence of a plasma, can create electron–hole pairs and oxygen vacancies, even if ultraviolet (UV) emissions in the presence of plasma are insignificant.^8,33,34^ In CO_2_ photocatalysis, vacancies are postulated as active sites.^35^ Consequently, reducible oxides with plausibly higher vacancies have been proposed for plasma-catalytic CO_2_ dissociation.^36^ Indeed, O atoms formed upon CO_2_ dissociation in the gas phase (e.g., via electron impact reactions) can potentially impinge on the catalytic surface (before recombining in the gas phase) to dislodge a surface oxygen atom and, thereby, create a vacancy (i.e., O­(g) + TiO_2_ → O_2_(g) + TiO_2v_). Calculations based on reported ab initio vacancy formation energy^37^ and standard thermodynamic tables for heat of formation of gaseous O atom show that this step is exothermic (∼−194 kJ/mol based on ab initio computed energy). Reported density functional theory (DFT) calculations show that once vacancies are formed on rutile or anatase phases of TiO_2_, CO_2_ can dissociate on the vacancy, thereby filling it with oxygen. Indeed, on (i) the 001 facet of anatase TiO_2_ with an oxygen vacancy,^38^ the CO_2_ binding energy is ∼−200 kJ/mol and the subsequent C–O dissociation barrier is ∼80 kJ/mol (relative to ∼450 kJ/mol on defect-free surface); (ii) the 101 facet of anatase TiO_2_ with an oxygen vacancy,^39^ the CO_2_ binding energy is ∼−120 kJ/mol with activation barriers between 45 and 100 kJ/mol depending on the site; (iii) 110 facet of rutile TiO_2_ with an oxygen vacancy,^40^ the CO_2_ binding energy is ∼−50 kJ/mol, while the dissociation enthalpy of adsorbed CO_2_ to CO and vacancy-filled TiO_2_ is ∼8 kJ/mol.^37^ Furthermore, scanning tunneling microscopy results show that CO_2_ can occupy surface vacancies on TiO_2_,^41^ dissociate to form CO and repair the surface defect,^42^ and enable vacancies to migrate.^43^

Such a coupled gas-surface mechanism involving the formation of oxygen atoms in the gas phase, creation of surface vacancies through oxygen abstraction, and facile dissociation of CO_2_ on the resultant vacancy has been proposed for microwave discharge plasma catalysis.^44^ However, these oxygen vacancies would also allow molecular oxygen (O_2_) to bind competitively with CO_2_ (the computed binding energy is −200 kJ/mol^37^ on a surface vacancy on rutile TiO_2_ (110) and even larger on anatase TiO_2_ (101)); therefore, from a thermodynamic standpoint, cofeeding O_2_ should lead to a decrease in surface coverage of adsorbed CO_2_, thereby lowering the rate of C–O dissociation. That is, cofeeding O_2_ should inhibit catalytic activity. However, as shown in Figure 6, the gas-phase conversion increases with O_2_ cofeed while surface contribution (inferred from the difference between conversion/rate with and without a catalyst) is independent of O_2_ cofeed. This indicates that surface vacancies are either not present or do not play a role in surface reactions.

**Figure 6:**
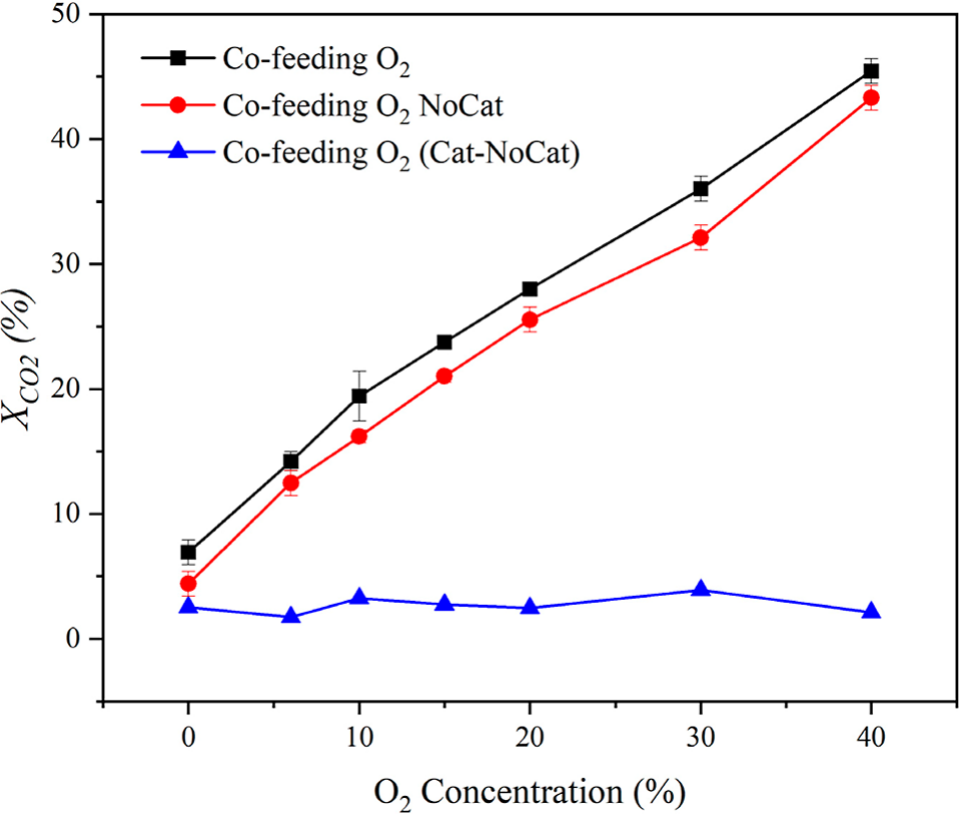
CO_2_ conversion versus concentration of oxygen. All other input factors are fixed. The flow rate is 70 ccm, the discharge power is 35 W, and the temperature is 140 °C. “Cat” and “NoCat” refer to runs with and without a catalyst, respectively. “Cat-NoCat” refers to the difference computed between these two sets of runs for the same experimental conditions.

Prior DFT calculations^38,39^ report high barriers (350–440 kJ/mol) for CO_2_ dissociation on pristine TiO_2_ surfaces (i.e., in the absence of defects); these values (even after correcting for CO_2_ physisorption) are significantly higher than the apparent barrier obtained from the model. However, there are two possibilities to explain this discrepancy. First, high-energy vibrationally excited CO_2_, which can be formed in the gas phase in the presence of plasma,^28^ will have a lower activation barrier for C–O scission on the surface; such arguments have been made for other plasma-catalysis systems such as ammonia synthesis and methane reforming.^45^ The extent of vibrational excitation will depend on the plasma power and thereby on SEI (hence, our results show a nonzero *E*_SEI_ for the surface reaction). Second, plasma can induce charge accumulation on the surface, the extent of which can depend on the field strength (also a function of plasma power and, thereby, SEI); this can further aid in the adsorption of molecules such as CO_2_ ^46–49^ and, plausibly, subsequent dissociation.^42^ Both cases can lead to the observed first-order dependence on CO_2_, especially if its surface coverage remains low.

The increase in conversion with the O_2_ concentration (Figure 6) is counterintuitive from a thermodynamic standpoint, especially at higher conversion, and in view of the reverse (recombination) reaction between CO and O. However, observations can be rationalized in many ways. The vibrationally assisted CO_2_ splitting in dielectric barrier discharges plays a central role in CO_2_ dissociation.^50^ In particular, the vibrational excitation of O_2_ is especially relevant, as O_2_ dissociation generates atomic oxygen that can subsequently react with CO_2_ molecules, thereby enhancing the overall conversion.^50^ In addition, the presence of O_2_ may contribute to moderating local gas temperatures by absorbing part of the plasma-generated energy, which can suppress reverse reactions associated with CO_2_ recombination.^23^ Collectively, these findings support the conclusion that the observed enhancement in CO_2_ conversion with O_2_ cofeed is dominated by gas-phase plasma effects, while the surface contribution remains largely unaffected by O_2_ concentration.^51^

### 3.6 Analysis of Model Predictions

#### 3.6.1 Contour Plots

Figure 7 shows contour plots of (I) the rate of CO_2_ conversion ( $R_{CO_{2}}$ ), (II) total energy efficiency, and (III) gas-phase contributions (ζ) with respect to input parameters to understand model trends and the effect of operating conditions. Similar plots of conversion with and without a catalyst and the energy intensity are in the Supporting Information (Figures SI1 and SI2). We discuss several observations below:

1.
$R_{CO_{2}}$ increases with the gas flow rate at a given inlet concentration, which directly follows from its definition (eq 8); it also increases with plasma power (i.e., SEI) at a fixed inlet concentration due to improved kinetics (and hence conversion); at a given flow rate or plasma power, on the other hand, $R_{CO_{2}}$ initially increases with inlet CO_2_ concentration (because of the definition) but then starts falling because conversion drops (as kinetics drops with reducing Ar concentration), thereby reducing the net amount of CO_2_ processed.
2. The total energy efficiency varies from 0.4 to 2% in the range of conditions explored. The range is consistent with some prior work^52^ but smaller than others.^53^ We reiterate that the thermal energy required to heat the reactor to maintain the reaction temperature; in scaled-up processes, the reactor heating requirements (per unit kg of CO_2_ processed) are expected to be more moderate and can potentially be met through heat integration. Therefore, if only plasma power is considered, the plasma energy efficiency (EE_plasma_) varies from 3 to 12%.
3. The total energy efficiency increases with flow rate and plasma power for a given inlet concentration. This is similar to $R_{CO_{2}}$ since, by definition, the efficiency depends on the rate at which CO_2_ is converted. At a given inlet concentration, an increase in temperature decreases the total energy efficiency even though surface reaction kinetics are expected to increase; this is because the heating power increases faster than the conversion due to the increased rate. At a given flow rate, temperature, and plasma power, the total energy efficiency first increases with respect to CO_2_ concentration to reach a maximum and then drops.
4. The gas-phase reactions are dominant across the parameter space (>65%) and particularly high (>90%) in large parts of the space. At a given plasma power, flow rate, or temperature, the gas-phase contribution drops with the CO_2_ inlet concentration. Surface contribution, as a fraction of the overall rate (i.e., 1 – ζ), peaks at nearly pure CO_2_ feed, high temperature, and low plasma power, which is evident from the learned kinetics expressions in Section 3.4.
5. In contrast to total energy efficiency, temperature only mildly influences $R_{CO_{2}}$ as conversion is largely due to gas-phase reactions;, therefore, contour lines remain vertical at lower inlet concentrations, while tilting at nearly pure CO_2_ feed, where catalyst contributions are at their highest and are dependent on temperature.
6. The maximum in most plots is at the bounds; however, for energy efficiency and $R_{CO_{2}}$ , the maximum is at an intermediate CO_2_ inlet concentration due to the interplay of the first-order kinetics and the effect of dilution.

**Figure 7:**
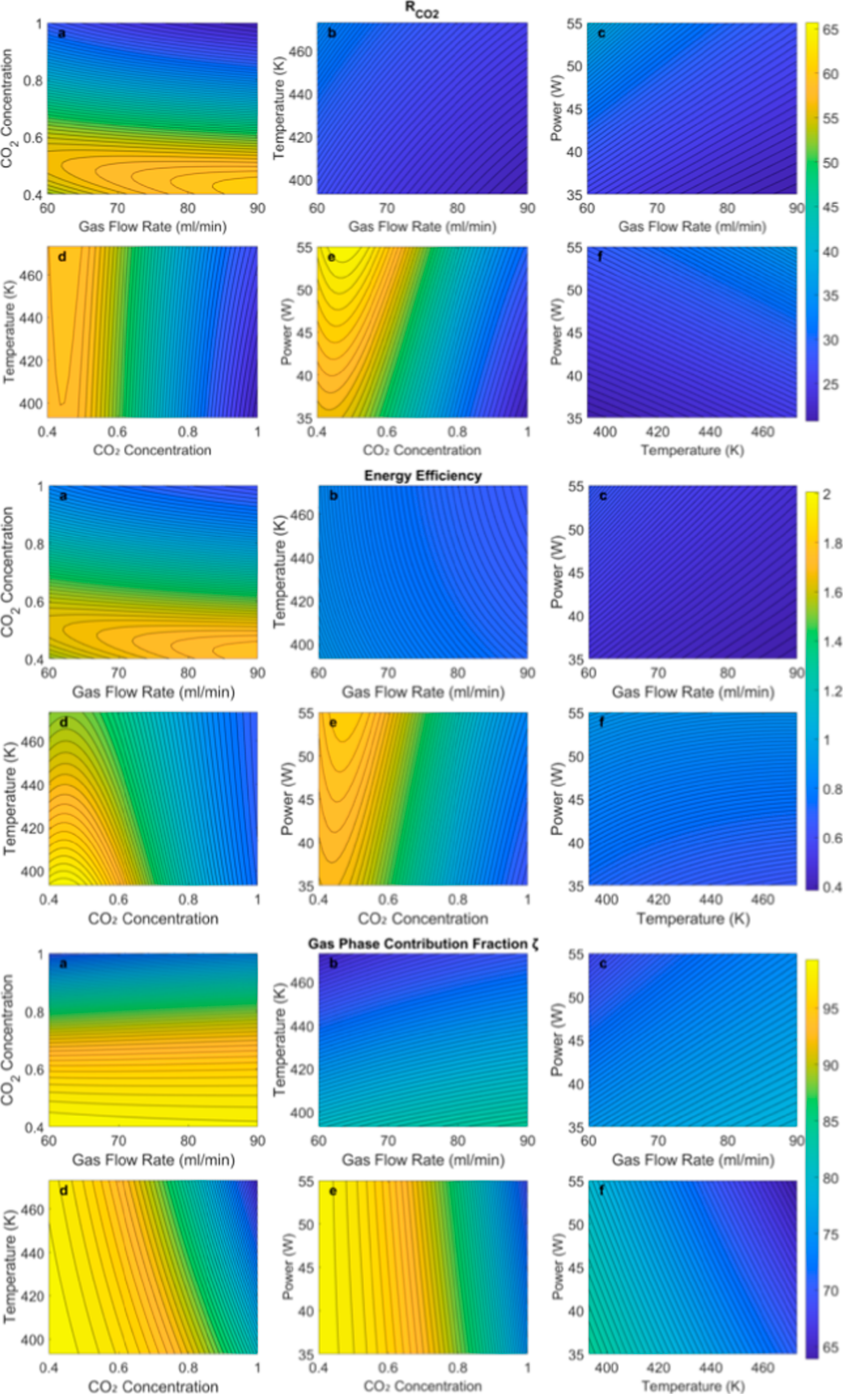
Contour plots of $R_{CO_{2}}$ , energy efficiency (EE_total_), and ζ for varying (a) CO_2_ concentration and gas flow rate, (b) temperature and gas flow rate, (c) plasma power and gas flow rate, (d) temperature and CO_2_ concentration, (e) plasma power and CO_2_ concentration, (d) plasma power and temperature. The CO_2_ concentration is varied from 0.4 to 1 (40–100%), the plasma power from 35 to 55 W, the temperature in the range of 393.15 K–473.15 K, and the flow rates from 60 to 90 mL/min. The nominal values used for inputs that were fixed were according to the central point of the Box–BBD: 433.15 K for temperature, 97% for CO_2_ concentration, 77.5 mL/min for total flow rate, and 42.5 W for plasma power. The details of the process of making these plots can be found in Section 2.1 in Supporting Information.

### 3.7 Optimization of Total Energy Efficiency

A brute force search was carried out to find the conditions that maximize the total energy efficiency (the details are shown in Section 2.2 in Supporting Information). The results are given in Table 4.

**Table 4:** Operating Conditions to Maximize Energy Efficiency

energy efficiency (%)	flow rate (mL/min)	CO_2_ concentration (%)	temperature (K)	plasma power (W)	CO_2_ conversion (%)
2.07	90	45	393.15	55	70.73

The optimal values from the model give an intermediate CO_2_ feed concentration (∼45%), a high flow rate (90 mL/min), a low temperature (393.15 K), and a high plasma power (55 W) to achieve a conversion of ∼71% and an energy efficiency of 2.1% (the plasma energy efficiency alone is ∼8.7%, which is lower than the maximum plasma efficiency found in the contour plots). The high optimal conversion could be an overprediction due to assumptions made in the model; the assumption of no reverse reaction or volume expansion upon reaction can lead to higher predicted conversion, while neglecting the effect of O_2_ (which is found to be positive) compensates through underprediction. However, it should be noted that per-pass conversion >50% has been reported on other catalysts, although without extensive optimization.^54,55^

### 3.8 Potential for Catalyst Design

The energy efficiency (even using the electrical energy basis) that we find for the plasma system is considerably lower than that for electrochemical CO_2_ conversion, either low-temperature (up to 60%)^53,56–58^ or high-temperature (∼90%) processes.^59,60^ One plausible way to improve energy efficiency in a plasma catalytic system is via reaction engineering strategies, e.g., through pulsing.^55^ Alternatively, judicious selection of the catalyst with apparent activation barriers lower than those of TiO_2_ can lead to higher rates and, thereby, efficiencies. While the focus of this work is on TiO_2_, the sensitivity of the model predictions to variation in *E*_a_ can be computed to evaluate the potential to improve the energy efficiency for this reaction through optimal catalyst selection. The best total (or electrical) energy efficiency can be as high as 4.3% (or 25%), as shown in Figure 8, if the apparent barrier is reduced to 5 kJ/mol (i.e., a reduction of ∼10 kJ/mol), underlining the potential impact of improved catalysts.

**Figure 8:**
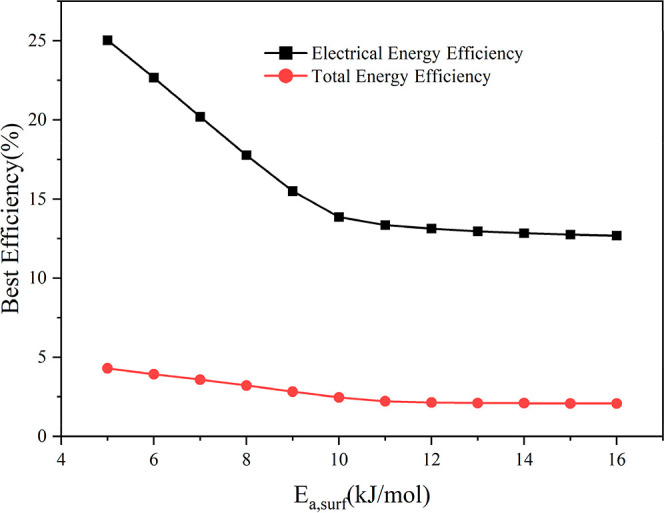
Best efficiency (given as a fraction) at different *E*_a,surf_ varying from 5 to 16 kJ/mol with a step of 1 kJ/mol. The range of operating conditions: the CO_2_ concentration is varied from 0.4 to 1 (40–100%), the plasma power from 35 to 55 W, the temperature in the range of 393.15 K–473.15 K, and the flow rates from 60 to 90 mL/min.

## 4 Conclusions

The kinetics of plasma-catalytic conversion of CO_2_ on TiO_2_ were evaluated rigorously through extensive experimentation and were accompanied by kinetic modeling. Our results indicate that while gas-phase reactions such as electron-impact dissociation of CO_2_ are dominant, resulting in first-order behavior, TiO_2_ can increase conversion by up to 35% depending on the processing conditions. Co-feeding Ar increases the kinetics; however, a larger cofeed fraction (>50 mol % in the feed) can reduce the total amount of CO_2_ converted (i.e., yield of CO) and lower the energy efficiency. Further evaluation of surface kinetic parameters, analysis of energetics using literature DFT data, and O_2_ cofeed studies revealed that surface oxygen vacancies may not play a significant role and that either a vibrationally excited CO_2_ dissociates in a facile manner on the surface or plasma-induced surface charging of TiO_2_ improves the binding strength of CO_2_ and possibly further dissociation. Total energy efficiency is limited by the heating power supplied in our experiments; however, a process with effective heat integration could lead to electrical energy efficiencies of up to 12%. While this is still lower than the reported electrical energy efficiency in the electrochemical conversion of CO_2_, there remains significant scope for improving efficiency through catalyst design.

## Supplementary Material





## Data Availability

Model simulations were implemented in *MATLAB R2024b* (The MathWorks, Inc., Natick, Massachusetts, USA) using custom scripts developed by the authors, which are provided as part of the extended Supporting Information.

## Notes

CBDBD CO_2_ split model and data (ZIP)
Model formulation, solution procedure, experimental data, carbon balance data, and a method to evaluate the sensitivity of the surface apparent barrier on overall efficiency (PDF)

## References

[1] Tommasi M., Degerli S. N., Ramis G., Rossetti I. (2024). Advancements in CO2Methanation:
A Comprehensive Review of Catalysis, Reactor Design and Process Optimization. Chem. Eng. Res. Des..

[2] Hanson E., Nwakile C., Hammed V. O. (2025). Carbon
Capture, Utilization, and
Storage (CCUS) Technologies: Evaluating the Effectiveness of Advanced
CCUS Solutions for Reducing CO2 Emissions. Results
Surf. Interf..

[3] George A., Shen B., Craven M., Wang Y., Kang D., Wu C., Tu X. (2021). A Review of
Non-Thermal Plasma Technology: A Novel
Solution for CO2 Conversion and Utilization. Renewable Sustainable Energy Rev..

[4] Xu S., Chen H., Hardacre C., Fan X. (2021). Non-Thermal Plasma
Catalysis for CO2conversion and Catalyst Design for the Process. J. Phys. D:Appl. Phys..

[5] Marcantonio V., De Falco M., Bocci E. (2022). Non-Thermal Plasma Technology for
CO2 Conversion—An Overview of the Most Relevant Experimental
Results and Kinetic Models. Energies.

[6] Gao Y., Zhou R., Chen B., Xiao L., Zhao X., Sun J., Zhou R., Zhang J., Liu Z. (2024). Plasma Catalysis-Driven
Decomposition of CO2: Optimizing Energy Distribution with NiCo-CuO
Catalyst. ACS Sustainable Chem. Eng..

[7] Indarto A., Choi J. W., Lee H., Song H. K. (2008). Decomposition of
Greenhouse Gases by Plasma. Environ. Chem. Lett..

[8] Mei D., Zhu X., Wu C., Ashford B., Williams P. T., Tu X. (2016). Plasma-Photocatalytic
Conversion of CO2 at Low Temperatures: Understanding the Synergistic
Effect of Plasma-Catalysis. Appl. Catal., B.

[9] Bogaerts A., Kozák T., Van Laer K., Snoeckx R. (2015). Plasma-Based Conversion
of CO2: Current Status and Future Challenges. Faraday Discuss..

[10] Chen G., Snyders R., Britun N. (2021). CO2conversion
Using Catalyst-Free
and Catalyst-Assisted Plasma-Processes: Recent Progress and Understanding. J. CO2 Util..

[11] Chung W. C., Chang M. B. (2016). Review of Catalysis
and Plasma Performance on Dry Reforming
of CH4 and Possible Synergistic Effects. Renewable
Sustainable Energy Rev..

[12] Gonzalez-Casamachin D.
A., Qin T., Huang W. M., Rangarajan S., Zhang L., Baltrusaitis J. (2024). Actively Learned
Optimal Sustainable Operation of Plasma-Catalyzed Methane Bireforming
on La0.7Ce0.3NiO3 Perovskite Catalyst. ACS Sustainable
Chem. Eng..

[13] Cao G., Gonzalez-Casamachin D. A., Xiao Y., Chen C. H., Uddi M., Baltrusaitis J. (2024). Evaluating
the Carbon Footprint of
the Integrated DBD-Plasma Bi-Reforming Unit via Laboratory Scale Experiments
and Scaled-up Process Modeling. Plasma Processes
Polym..

[14] Chi J., Wu X., Tao J., Min X., Li Z., Zhao S. (2024). DBD-Coupled
Highly Dispersed Ni/SiO2Materials for CO2 Reduction Performance and
Mechanism Study. J. Energy Inst..

[15] Tu X., Whitehead J. C. (2012). Plasma-Catalytic
Dry Reforming of Methane in an Atmospheric
Dielectric Barrier Discharge: Understanding the Synergistic Effect
at Low Temperature. Appl. Catal., B.

[16] Bacariza M. C., Graça I., Bebiano S. S., Lopes J. M., Henriques C. (2017). Magnesium
as Promoter of CO2Methanation on Ni-Based USY Zeolites. Energy Fuels.

[17] Ray D., Chawdhury P., Subrahmanyam C. (2020). A Facile Method to Decompose CO2
Using a G-C3N4-Assisted DBD Plasma Reactor. Environ. Res..

[18] Schneider J., Matsuoka M., Takeuchi M., Zhang J., Horiuchi Y., Anpo M., Bahnemann D. W. (2014). Understanding TiO2 Photocatalysis
Mechanisms and Materials. Chem. Rev..

[19] Jõgi I., Haljaste A., Laan M. (2014). Hybrid TiO2
Based Plasma-Catalytic
Reactors for the Removal of Hazardous Gasses. Surf. Coat. Technol..

[20] Huang Y., Ho S. S. H., Niu R., Xu L., Lu Y., Cao J., Lee S. (2016). Removal of Indoor Volatile
Organic Compounds via Photocatalytic
Oxidation: A Short Review and Prospect. Molecules.

[21] Tu X., Gallon H. J., Whitehead J. C. (2011). Electrical
and Spectroscopic Diagnostics
of a Single-Stage Plasma-Catalysis System: Effect of Packing with
TiO2. J. Phys. D:Appl. Phys..

[22] Xu S., Whitehead J. C., Martin P. A. (2017). CO2 Conversion in a Non-Thermal,
Barium Titanate Packed Bed Plasma Reactor: The Effect of Dilution
by Ar and N2. Chem. Eng. J..

[23] Zhang K., Zhang G., Liu X., Phan A. N., Luo K. (2017). A Study on
CO2 Decomposition to CO and O2 by the Combination of Catalysis and
Dielectric-Barrier Discharges at Low Temperatures and Ambient Pressure. Ind. Eng. Chem. Res..

[24] Zhang K., Harvey A. P. (2021). CO2 Decomposition to CO in the Presence
of up to 50%
O2 Using a Non-Thermal Plasma at Atmospheric Temperature and Pressure. Chem. Eng. J..

[25] Xu S., Khalaf P. I., Martin P. A., Whitehead J. C. (2018). CO2 Dissociation
in a Packed-Bed Plasma Reactor: Effects of Operating Conditions. Plasma Sources Sci. Technol..

[26] Ramakers M., Michielsen I., Aerts R., Meynen V., Bogaerts A. (2015). Effect of
Argon or Helium on the CO2 Conversion in a Dielectric Barrier Discharge. Plasma Processes Polym..

[27] Ray D., Saha R., Subrahmanyam C. (2017). DBD Plasma Assisted CO2 Decomposition:
Influence of Diluent Gases. Catalysts.

[28] Aerts R., Martens T., Bogaerts A. (2012). Influence of Vibrational
States on
CO2 Splitting by Dielectric Barrier Discharges. J. Phys. Chem. C.

[29] Bogaerts A., Neyts E. C. (2018). Plasma Technology: An Emerging Technology for Energy
Storage. ACS Energy Lett..

[30] Snoeckx R., Bogaerts A. (2017). Plasma Technology-a
Novel Solution for CO2 Conversion?. Chem. Soc.
Rev..

[31] Hegemann D. (2023). Plasma Activation
Mechanisms Governed by Specific Energy Input: Potential and Perspectives. Plasma Processes Polym..

[32] Aerts R., Somers W., Bogaerts A. (2015). Carbon Dioxide Splitting in a Dielectric
Barrier Discharge Plasma: A Combined Experimental and Computational
Study. ChemSusChem.

[33] Nakamura I., Negishi N., Kutsuna S., Ihara T., Sugihara S., Takeuchi K. (2000). Role of Oxygen Vacancy in the Plasma-Treated
TiO2 Photocatalyst
with Visible Light Activity for NO Removal. J. Mol. Catal. A:Chem..

[34] Whitehead J. C. (2010). Plasma
Catalysis: A Solution for Environmental Problems. Pure Appl. Chem..

[35] Ji Y., Luo Y. (2016). New Mechanism for Photocatalytic Reduction of CO2 on
the Anatase
TiO2(101) Surface: The Essential Role of Oxygen Vacancy. J. Am. Chem. Soc..

[36] Ashford B., Wang Y., Poh C. K., Chen L., Tu X. (2020). Plasma-Catalytic
Conversion of CO2 to CO over Binary Metal Oxide Catalysts at Low Temperatures. Appl. Catal., B.

[37] Hinuma Y., Toyao T., Kamachi T., Maeno Z., Takakusagi S., Furukawa S., Takigawa I., Shimizu K. I. (2018). Density Functional
Theory Calculations of Oxygen Vacancy Formation and Subsequent Molecular
Adsorption on Oxide Surfaces. J. Phys. Chem.
C.

[38] Huygh S., Bogaerts A., Neyts E. C. (2016). How Oxygen
Vacancies Activate CO2
Dissociation on TiO2 Anatase (001). J. Phys.
Chem. C.

[39] Sorescu D. C., Al-Saidi W. A., Jordan K. D. (2011). CO2 Adsorption on TiO2(101) Anatase:
A Dispersion-Corrected Density Functional Theory Study. J. Chem. Phys..

[40] Sorescu D.
C., Lee J., Al-Saidi W. A., Jordan K. D. (2011). CO2 Adsorption on TiO2(110) Rutile:
Insight from Dispersion-Corrected Density Functional Theory Calculations
and Scanning Tunneling Microscopy Experiments. J. Chem. Phys..

[41] Lee J., Sorescu D. C., Deng X., Jordan K. D. (2011). Diffusion of CO
2 on the Rutile TiO 2(110) Surface. J. Phys.
Chem. Lett..

[42] Lee J., Sorescu D. C., Deng X. (2011). Electron-Induced
Dissociation of
CO 2 on TiO 2 (110). J. Am. Chem. Soc..

[43] Kim Y. J., Choi H., Kim D., Kim Y., Kim K.-j., Kim J., Thornton G., Kim H. Y., Park J. Y. (2025). CO2-Driven Oxygen
Vacancy Diffusion and Healing on TiO2(110) at Ambient Pressure. Angew. Chem., Int. Ed..

[44] Chen G., Britun N., Godfroid T., Georgieva V., Snyders R., Delplancke-Ogletree M.
P. (2017). An Overview of CO2 Conversion
in a Microwave Discharge: The Role of Plasma-Catalysis. J. Phys. D:Appl. Phys..

[45] Kim J., Go D. B., Hicks J. C. (2017). Synergistic Effects of Plasma-Catalyst
Interactions for CH4 Activation. Phys. Chem.
Chem. Phys..

[46] Doherty F., Goldsmith B. R. (2023). Modeling
Plasma-Induced Surface Charge Effects on CO2
Activation by Single Atom Catalysts Supported on Reducible and Irreducible
Metal Oxides. Plasma Sources Sci. Technol..

[47] Bal K. M., Huygh S., Bogaerts A., Neyts E. C. (2018). Effect
of Plasma-Induced
Surface Charging on Catalytic Processes: Application to CO2 Activation. Plasma Sources Sci. Technol..

[48] Deskins N. A., Rousseau R., Dupuis M. (2010). Defining the Role of
Excess Electrons
in the Surface Chemistry of TiO 2. J. Phys.
Chem. C.

[49] Yin W. J., Wen B., Bandaru S., Krack M., Lau M. W., Liu L. M. (2016). The Effect
of Excess Electron and Hole on CO2 Adsorption and Activation on Rutile
(110) Surface. Sci. Rep..

[50] Bogaerts A., Berthelot A., Heijkers S., Kolev S., Snoeckx R., Sun S., Trenchev G., Van Laer K., Wang W. (2017). CO2 Conversion by Plasma
Technology: Insights from Modeling the Plasma Chemistry and Plasma
Reactor Design. Plasma Sources Sci. Technol..

[51] Kolb T., Voigt J. H., Gericke K. H. (2013). Conversion of Methane
and Carbon
Dioxide in a DBD Reactor: Influence of Oxygen. Plasma Chem. Plasma Process..

[52] Mei D., Tu X. (2017). Atmospheric Pressure Non-Thermal Plasma Activation
of CO2 in a Packed-Bed
Dielectric Barrier Discharge Reactor. ChemPhysChem.

[53] Ozkan A., Bogaerts A., Reniers F. (2017). Routes to
Increase the Conversion
and the Energy Efficiency in the Splitting of CO2 by a Dielectric
Barrier Discharge. J. Phys. D:Appl. Phys..

[54] Umeojiakor C., Toghiani H., Xiang Y. (2025). Enhancing CO 2 Conversion
through
Plasma Catalytic Synergy under Subcooled Conditions. Ind. Eng. Chem. Res..

[55] Li J., Zhu S., Lu K., Ma C., Yang D., Yu F. (2021). CO2 Conversion
in a Coaxial Dielectric Barrier Discharge Plasma Reactor in the Presence
of Mixed ZrO2-CeO2. J. Environ. Chem. Eng..

[56] Reichbauer T., Schmid B., Vetter K. M., Reinisch D., Martić N., Reller C., Grasruck A., Dorta R., Schmid G. (2024). Electrical
Energy Input Efficiency Limitations in CO2-to-CO Electrolysis and
Attempts for Improvement. Electrochem. Sci.
Adv..

[57] Zhong S., Sui P., Holtappels P., Navarrete A., Li F., Dittmeyer R. (2025). Robust and
Efficient Electroreduction of CO2 to CO in a Modified Zero-Gap Electrochemical
Cell. Chem. Eng. J..

[58] Lee H., Kwon S., Park N., Cha S. G., Lee E., Kong T. H., Cha J., Kwon Y. (2024). Scalable Low-Temperature
CO2 Electrolysis: Current Status and Outlook. JACS Au.

[59] Lu L., Liu W., Wang J., Wang Y., Xia C., Zhou X. D., Chen M., Guan W. (2020). Long-Term Stability of Carbon Dioxide
Electrolysis in a Large-Scale Flat-Tube Solid Oxide Electrolysis Cell
Based on Double-Sided Air Electrodes. Appl.
Energy.

[60] Wu A., Li C., Han B., Liu W., Zhang Y., Hanson S., Guan W., Singhal S. C. (2023). Pulsed
Electrolysis of Carbon Dioxide
by Large-Scale Solid Oxide Electrolytic Cells for Intermittent Renewable
Energy Storage. Carbon Energy.

