# Supplementary material for: Mechanistic Insights for Plasma-Catalytic CO2 Reduction over TiO2 in a Dielectric Barrier Discharge Reactor
Source: ACS Eng Au. 2026 Jan 27;6(2):334–44. doi: 10.1021/acsengineeringau.5c00092 (PMC13088181; doi:10.1021/acsengineeringau.5c00092)
Supplement: Supplementary file 2 [file eg5c00092_si_002.pdf]

Supporting Information

Mechanistic Insights for Plasma-Catalytic CO_2 Reduction over TiO_2 in Dielectric Barrier Discharge Reactor

Diego Alexander Gonzalez-Casamachin, Yuyang Hu, Srinivas Rangarajan,* and Jonas Baltrusaitis*

Department of Chemical and Biomolecular Engineering, Lehigh University, 111 Research Dr., Bethlehem, PA 18015, USA.

1.1 Mathematical model

From the view of reaction engineering, the reactor can be seen as a PFR (plug flow reactor), whose material balance equation can be written as follows:

$$\frac{dX_A}{d\tau} = \frac{-r_A}{C_{A0}}$$

In our research work, we separately conducted the experiments with/without catalysts in order to understand the kinetics characteristics of plasma alone and when combined with catalysts. So we developed a simple kinetics model to describe the gas phase kinetics and surface kinetics as Eqs. (2) to (5),

$$r_A = k_g {C_A}^m + k_s {C_A}^n \ (wi/$$

$$r_A = k_g {C_A}^m$$

$$k_g = A_g e^{-\frac{E_{a,g}}{RT}} * e^{SEI*E_{SEI,g}} * e^{\beta * C_{Ar}(\%)} 4)$$

$$k_s = A_s e^{-\frac{E_{a,s}}{RT}} * e^{SEI*E_{SEI,s}} 5)$$

$$\tau = \frac{V_R}{\upsilon_0} 6)$$

τ is the space time, V_R is the volume of reactor and υ_0 is the volume flow rate. In fact, this is a reaction where there is an increase in the number of moles with reaction, but given the reaction conversion in our experiment ranges from 5% - 23%, so the change of volume is negligible. This model briefly contains all the variables in our experiment. After organizing these formulas above, we can get the following differential equation as Eq. (7)

$$\frac{d(1 - X_A)}{d\tau} = - k_g {C_{A0}}^{m-1} (1 - X_A)^m - k_s {C_{A0}}^{n-1} (1 - X_A)^n$$

The formulae to calculate objective function (*OF*) and mean relative error (*MRE*) are shown in Eqs. (8) and (9), the best parameters were gained by minimizing the objective function.

$$OF = \sum_{i=1}^{n} (X_A^{cal} - X_A^{exp})^2 (8)$$

$$MRE = \frac{1}{N} \sum_{i=1}^{N} \left| \frac{X_A^{cal} - X_A^{exp}}{X_A^{exp}} \right| (9)$$

1.2 Solution procedure for parameter estimation

In consideration that there are two phases (gas and surface), the estimation of parameters in each phase was carried individually. The whole process was as follows:

(1) The order m and n were first assumed to equal to 1 while the other parameters were obtained by minimizing the objective function. Various integral orders were considered, however, m=n=1 gave the best results.

(2) Latin Hypercube Sampling method was adopted to generate a large amount of evenly distributed initial values of parameters within the specified range. These serve as initial guesses during parameter estimation.

(3) The *ode45* and *ode15s* were applied to solve Eq. (7) while *lsqnonlin* was applied to optimize the parameters in the model by nonlinear least square method for each initial guess. An approximate boundary of each parameter was found by iterating in the similar process.

(4) Besides the methods above, the mean relative error (*MRE*) and adjusted R^2 were also calculated to evaluate the validity of the model.

The detailed code can be seen in the attached files multiple_regression.m and its 2 function files residual_no_catalyst.m and residual_catalyst.m which solves the kinetic differential equation in the situation without catalyst and with catalyst individually.

1.3 Experimental data

The data used for the regression are shown in Table SI1.

Table SI1 Experimental result of CO_2 decomposition with and without TiO_2 catalyst

run	Discharge Power(W)	Temperature (°C)	CO_2 Concentration	Flow rate (ml/min)	Conversion Without Cat	Conversion With Cat
1	35	140	1	70	4.94%	7.43%
2	35	140	0.98	70	6.61%	9.13%
3	35	140	0.96	70	9.34%	11.05%
4	35	140	0.94	70	10.60%	12.43%
5	35	150	1	70	5.68%	7.55%
6	35	150	0.98	70	6.65%	9.82%

7	35	150	0.96	70	9.23%	11.44%
8	35	150	0.94	70	10.93%	12.49%
9	35	160	1	70	5.69%	8.75%
10	35	160	0.98	70	7.40%	10.42%
11	35	160	0.96	70	9.31%	11.87%
12	35	160	0.94	70	10.85%	13.19%
13	35	170	1	70	5.69%	9.08%
14	35	170	0.98	70	7.52%	10.63%
15	35	170	0.96	70	9.47%	11.92%
16	35	170	0.94	70	10.99%	14.09%
17	40	140	1	70	/	9.10%
18	40	140	0.98	70	/	11.11%
19	40	140	0.96	70	/	12.95%
20	40	140	0.94	70	/	13.69%
21	40	150	1	70	/	9.79%
22	40	150	0.98	70	/	11.74%
23	40	150	0.96	70	/	13.28%
24	40	150	0.94	70	/	14.41%
25	40	160	1	70	/	9.79%
26	40	160	0.98	70	/	11.80%
27	40	160	0.96	70	/	13.49%
28	40	160	0.94	70	/	15.41%
29	40	170	1	70	/	10.78%
30	40	170	0.98	70	/	11.87%
31	40	170	0.96	70	/	13.67%
32	40	170	0.94	70	/	15.22%
33	45	140	1	70	/	12.68%
34	45	140	0.98	70	/	14.78%
35	45	140	0.96	70	/	16.39%
36	45	140	0.94	70	/	18.41%
37	45	150	1	70	/	12.74%
38	45	150	0.98	70	/	14.80%
39	45	150	0.96	70	/	16.85%
40	45	150	0.94	70	/	18.86%
41	45	160	1	70	/	13.69%
42	45	160	0.98	70	/	16.10%
43	45	160	0.96	70	/	18.59%
44	45	160	0.94	70	/	19.49%
45	45	170	1	70	/	14.14%
46	45	170	0.98	70	/	16.27%
47	45	170	0.96	70	/	18.89%
48	45	170	0.94	70	/	19.72%
49	50	140	1	70	/	16.10%
50	50	140	0.98	70	/	18.29%

51	50	140	0.96	70	/	20.20%
52	50	140	0.94	70	/	21.71%
53	50	150	1	70	/	16.52%
54	50	150	0.98	70	/	18.41%
55	50	150	0.96	70	/	20.35%
56	50	150	0.94	70	/	21.76%
57	50	160	1	70	/	18.92%
58	50	160	0.98	70	/	19.75%
59	50	160	0.96	70	/	22.06%
60	50	160	0.94	70	/	22.72%
61	50	170	1	70	/	19.27%
62	50	170	0.98	70	/	20.05%
63	50	170	0.96	70	/	22.07%
64	50	170	0.94	70	/	22.84%
65	35	140	0.97	77.5	6.65%	9.53%
66	50	140	0.97	77.5	15.02%	19.73%
67	42.5	140	0.94	77.5	9.59%	17.87%
68	42.5	140	1	77.5	12.62%	13.07%
69	42.5	140	0.97	70	11.09%	15.26%
70	42.5	140	0.97	85	14.03%	16.25%
71	42.5	155	0.94	70	15.38%	16.85%
72	42.5	155	1	70	10.37%	12.29%
73	42.5	155	0.94	85	13.64%	16.67%
74	42.5	155	1	85	8.03%	11.48%
75	35	155	0.97	70	11.00%	13.13%
76	50	155	0.97	70	14.96%	20.39%
77	35	155	0.97	85	8.99%	10.19%
78	50	155	0.97	85	15.65%	18.95%
79	35	155	0.94	77.5	12.11%	14.51%
80	50	155	0.94	77.5	18.11%	22.73%
81	35	155	1	77.5	6.05%	9.50%
82	50	155	1	77.5	12.59%	17.54%
83	42.5	155	0.97	77.5	11.99%	15.11%
84	42.5	155	0.97	77.5	12.08%	14.84%
85	42.5	155	0.97	77.5	12.29%	15.11%
86	35	170	0.97	77.5	9.65%	12.50%
87	50	170	0.97	77.5	15.59%	18.80%
88	42.5	170	0.94	77.5	13.97%	16.40%
89	42.5	170	1	77.5	8.48%	11.06%
90	42.5	170	0.97	70	11.96%	14.24%
91	42.5	170	0.97	85	10.37%	12.47%
92	35	140	1	75	/	6.04%
93	35	140	0.98	75	/	7.66%
94	35	140	0.96	75	/	9.56%

95	35	140	0.94	75	/	12.07%
96	35	150	1	75	/	7.31%
97	35	150	0.98	75	/	8.23%
98	35	150	0.96	75	/	10.24%
99	35	150	0.94	75	/	12.53%
100	35	160	1	75	/	9.13%
101	35	160	0.98	75	/	10.34%
102	35	160	0.96	75	/	12.76%
103	35	160	0.94	75	/	12.67%
104	35	170	1	75	/	9.17%
105	35	170	0.98	75	/	10.60%
106	35	170	0.96	75	/	12.38%
107	35	170	0.94	75	/	13.16%
108	40	140	1	75	/	12.40%
109	40	140	0.98	75	/	13.97%
110	40	140	0.96	75	/	15.77%
111	40	140	0.94	75	/	16.46%
112	40	150	1	75	/	12.76%
113	40	150	0.98	75	/	14.48%
114	40	150	0.96	75	/	16.46%
115	40	150	0.94	75	/	16.49%
116	40	160	1	75	/	13.07%
117	40	160	0.98	75	/	15.11%
118	40	160	0.96	75	/	16.81%
119	40	160	0.94	75	/	17.20%
120	40	170	1	75	/	13.36%
121	40	170	0.98	75	/	14.99%
122	40	170	0.96	75	/	16.88%
123	40	170	0.94	75	/	17.09%
124	45	140	1	75	/	12.17%
125	45	140	0.98	75	/	13.97%
126	45	140	0.96	75	/	16.36%
127	45	140	0.94	75	/	17.24%
128	45	150	1	75	/	12.40%
129	45	150	0.98	75	/	15.28%
130	45	150	0.96	75	/	16.49%
131	45	150	0.94	75	/	18.50%
132	45	160	1	75	/	12.86%
133	45	160	0.98	75	/	15.38%
134	45	160	0.96	75	/	16.72%
135	45	160	0.94	75	/	19.19%
136	45	170	1	75	/	13.45%
137	45	170	0.98	75	/	15.76%
138	45	170	0.96	75	/	16.93%

139	45	170	0.94	75	/	19.27%
140	50	140	1	75	/	16.34%
141	50	140	0.98	75	/	17.77%
142	50	140	0.96	75	/	19.40%
143	50	140	0.94	75	/	21.40%
144	50	150	1	75	/	16.79%
145	50	150	0.98	75	/	18.70%
146	50	150	0.96	75	/	19.33%
147	50	150	0.94	75	/	21.59%
148	50	160	1	75	/	16.91%
149	50	160	0.98	75	/	18.94%
150	50	160	0.96	75	/	19.55%
151	50	160	0.94	75	/	21.70%
152	50	170	1	75	/	16.97%
153	50	170	0.98	75	/	19.27%
154	50	170	0.96	75	/	19.93%
155	50	170	0.94	75	/	21.74%
156	35	140	1	80	/	5.30%
157	35	140	0.98	80	/	8.77%
158	35	140	0.96	80	/	10.58%
159	35	140	0.94	80	/	11.53%
160	35	150	1	80	/	5.30%
161	35	150	0.98	80	/	9.04%
162	35	150	0.96	80	/	10.88%
163	35	150	0.94	80	/	11.57%
164	35	160	1	80	/	7.01%
165	35	160	0.98	80	/	9.11%
166	35	160	0.96	80	/	10.93%
167	35	160	0.94	80	/	12.04%
168	35	170	1	80	/	7.30%
169	35	170	0.98	80	/	9.32%
170	35	170	0.96	80	/	10.96%
171	35	170	0.94	80	/	12.50%
172	35	140	0.8	70	22.94%	25.46%
173	45	140	0.8	70	24.44%	26.48%
174	35	140	0.8	70	28.55%	30.38%
175	45	140	0.8	70	31.64%	35.69%
176	55	140	0.8	70	33.32%	39.44%
177	65	140	0.8	70	4.40%	6.92%
178	75	140	0.8	70	8.93%	11.24%
179	35	140	1	70	12.26%	15.89%
180	45	140	1	70	15.35%	19.55%
181	55	140	1	70	17.06%	24.05%
182	65	140	1	70	12.44%	14.21%

183	75	140	1	70	16.19%	19.43%
184	35	140	0.94	70	21.02%	23.75%
185	35	140	0.9	70	25.55%	28.01%
186	35	140	0.85	70	32.12%	36.02%
187	35	140	0.8	70	43.34%	45.44%

Table SI2. Carbon balance of CO_2 conversion using different concentrations of Ar and O_2.

Diluent gas	Concentration	CO_2 Conversion (%)		Carbon Balance	
		With Catalyst	Without Catalyst	With Catalyst	Without Catalyst
O2	60%CO_2-40%O2	45.44	43.34	100.68%	99.31%
	70%CO2-30%O2	36.02	32.12	100.49%	96.32%
	80%CO2-20%O2	28.01	25.55	99.56%	99.92%
	85%CO2-15%O2	23.75	21.02	99.62%	100.07%
	90%CO2-10%O2	19.43	16.19	99.74%	99.83%
	94%CO2-6%O2	14.21	12.44	98.66%	99.99%
Ar	60%CO2-40%Ar	40.22	39.14	94.35%	94.73%
	70%CO2-30%Ar	33.29	31.4	95.33%	95.47%
	80%CO2-20%Ar	25.46	22.94	97.91%	96.93%
	85%CO2-15%Ar	21.23	18.95	96.89%	97.42%
	90%CO2-10%Ar	15.62	14.93	97.25%	97.94%
	94%CO2-6%Ar	13.445	11.81	96.43%	97.56%

Table SI3. Carbon balance of CO_2 conversion at different discharge powers.

	Discharge Power	CO_2 Conversion (%)	Carbon Balance

Without Diluent gas		With Catalyst	Without Catalyst	With Catalyst	Without Catalyst
Pure CO_2	35W-100%CO_2	6.92	4.4	98.47%	100.12%
	45W-100%CO_2	11.24	8.93	99.82%	99.82%
	55W-100%CO_2	15.89	12.26	99.60%	99.96%
	65W-100%CO_2	19.55	15.35	99.51%	99.83%
	75W-100%CO_2	24.05	17.06	99.32%	99.66%

Table SI4. Carbon balance of CO_2 conversion at different temperatures.

Compound	Temperature	CO2 Conversion (%)		Carbon Balance	
		With Catalyst	Without Catalyst	With Catalyst	Without Catalyst
Pure CO_2	140	7.43	4.94	97.50%	99.67%
	150	7.55	5.675	98.20%	99.32%
	160	8.75	5.69	96.52%	98.40%
	170	9.08	5.69	96.45%	97.34%

Table SI5. Process parameters and response of CO_2 plasma conversion

Run	Process parameters				Response			
	Discharge Power (W)	Temperature (°C)	CO_2 concentration (%)	Flow rate (mL/min)	Conversion Without Cat (%)	CB Without Cat (%)	Conversion With Cat (%)	CB With Cat (%)
1	35	140	97	77.5	6.7	100.00%	9.5	98.07%
2	50	140	97	77.5	15.0	95.13%	19.7	97.86%
3	42.5	140	94	77.5	9.6	97.74%	17.9	95.47%
4	42.5	140	100	77.5	12.6	97.80%	13.1	99.11%
5	42.5	140	97	70	11.1	95.14%	15.3	98.20%
6	42.5	140	97	85	14.0	99.59%	16.3	98.16%
7	42.5	155	94	70	15.4	95.29%	16.9	95.93%
8	42.5	155	100	70	10.4	100.02%	12.3	99.96%
9	42.5	155	94	85	13.6	97.82%	16.7	96.55%
10	42.5	155	100	85	8.0	97.84%	11.5	100.00%
11	35	155	97	70	11.0	97.72%	13.1	99.07%
12	50	155	97	70	15.0	96.50%	20.4	98.09%
13	35	155	97	85	9.0	99.55%	10.2	98.74%
14	50	155	97	85	15.7	95.38%	19.0	98.54%
15	35	155	94	77.5	12.1	97.78%	14.5	95.15%
16	50	155	94	77.5	18.1	100.06%	22.7	94.95%
17	35	155	100	77.5	6.1	95.61%	9.5	100.04%
18	50	155	100	77.5	12.6	95.81%	17.5	99.65%
19	42.5	155	97	77.5	12.0	100.08%	15.1	97.34%
20	42.5	155	97	77.5	12.1	98.86%	14.8	98.18%
21	42.5	155	97	77.5	12.3	97.73%	15.1	98.49%
22	35	170	97	77.5	9.7	97.76%	12.5	99.46%
23	50	170	97	77.5	15.6	97.77%	18.8	97.53%
24	42.5	170	94	77.5	14.0	98.30%	16.4	96.31%
25	42.5	170	100	77.5	8.5	98.02%	11.1	99.46%
26	42.5	170	97	70	12.0	97.97%	14.2	98.24%
27	42.5	170	97	85	10.37	97.51%	12.5	98.52%

2. Prediction of model

2.1 Contour plots

The kinetics data gained from programming above were applied to drawing contour plots. The overall process was as follows:

(1) The 4 adjustable parameters (flowrate, CO_2 concentration, temperature and plasma power) were given a range which is shown in Table SI.

Table SI6 Range of adjustable parameters

	Lower bound	Upper bound
Flowrate (ml/min)	60	90
CO_2 concentration	0.4	1
Temperature (K)	393.15	473.15
Plsam power (W)	35	55

Besides, the middle value in the range is nominal value for each parameter.

(2) The parameters were selected 50 data points evenly over the range. Each time, only 2 parameters were varied while the other 2 remained at the nominal value. So the combinations between different parameters should be 6. In each combination, all the system performance indicator (conversion, CO_2 converted amount per time, energy intensity and energy efficiency) are calculated at each point. Then the contour plots were drawn based on these calculation results

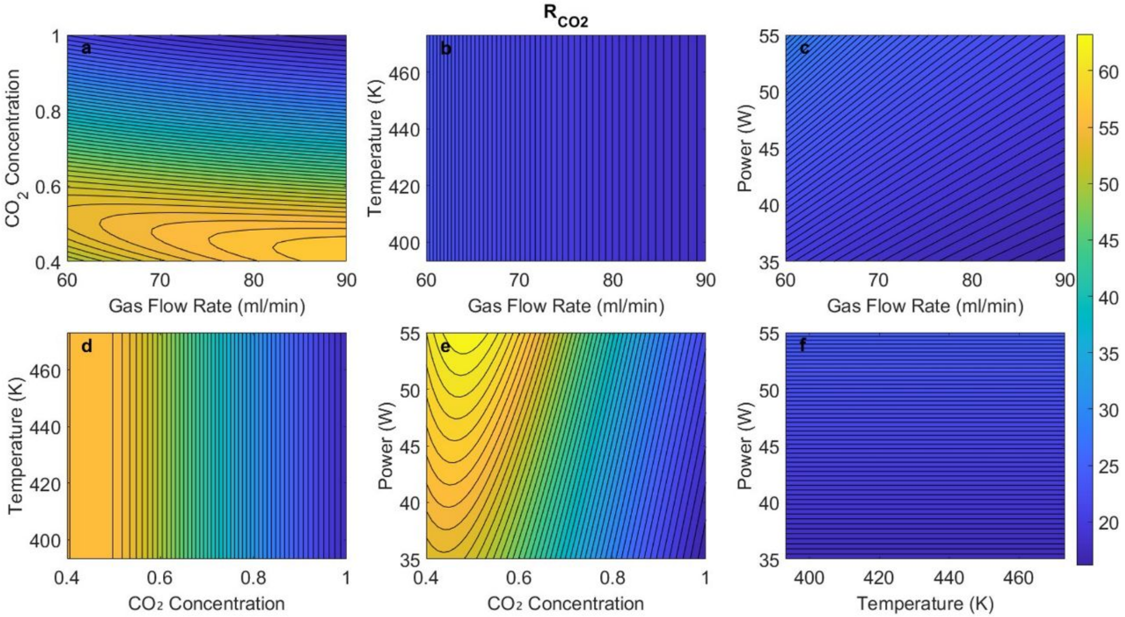
R CO2
a
b
c
d
e
f
CO2 Concentration
Gas Flow Rate (ml/min)
Temperature (K)
Power (W)
CO₂ Concentration
Temperature (K)

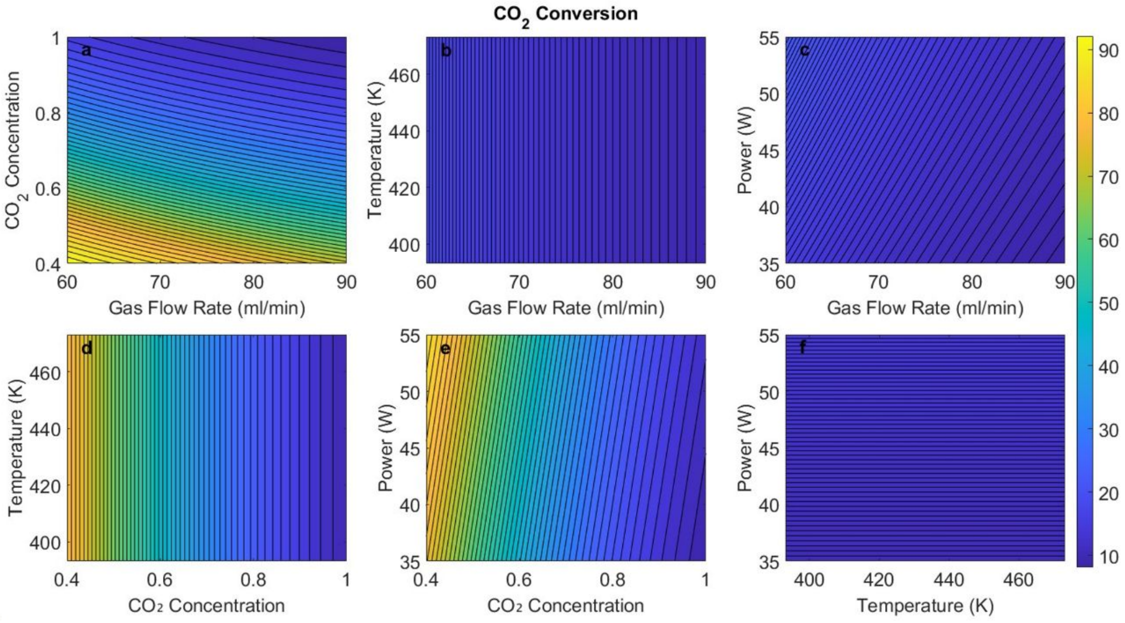
CO2 Conversion
a
b
c
d
e
f
CO2 Concentration
Gas Flow Rate (ml/min)
Temperature (K)
Power (W)
CO₂ Concentration
Temperature (K)

Figure SI1 Contour plots of different metric about reaction performance no cat: for varying (a) CO_2 concentration and gas flow rate (b) temperature and gas flow rate (c) plasma power and gas flowrate (d) temperature and CO_2 concentration (e) plasma power and CO_2 concentration (d) plasma power and temperature. All other parameters are set to their nominal values according to the central point of Box Behnken design: 428.15 K for temperature, 97% for CO2 concentration, 77.5 ml/min for total flow rate, and 42.5 W for plasma power.

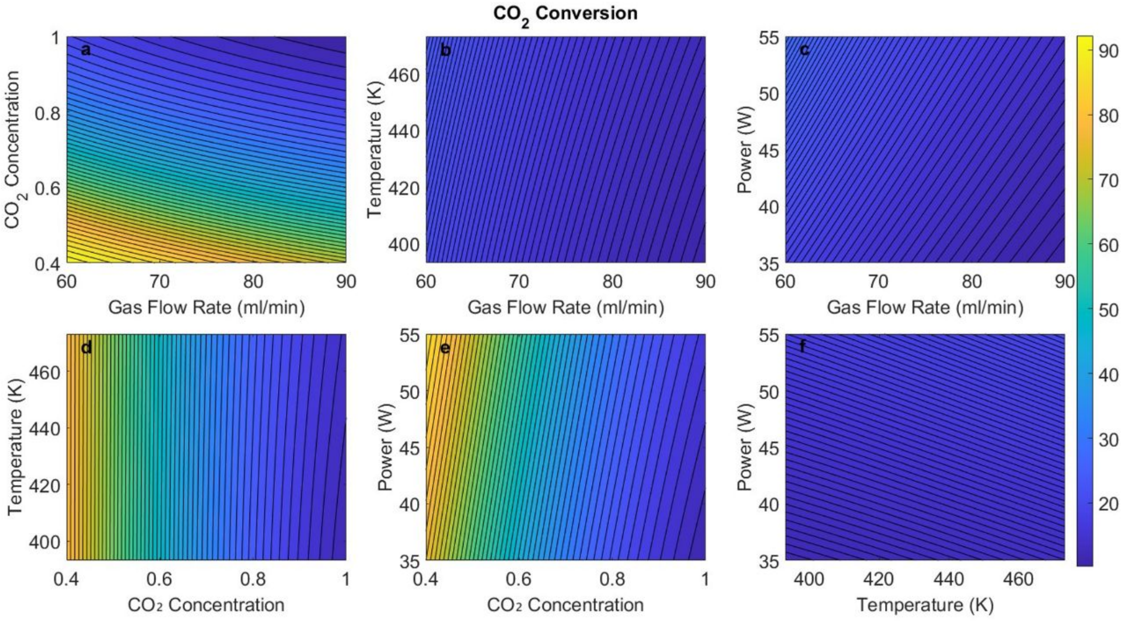
CO2 Conversion
a
b
c
d
e
f
CO2 Concentration
Gas Flow Rate (ml/min)
Temperature (K)
Power (W)
0.4
0.6
0.8
1
60
70
80
90
400
420
440
460
35
40
45
50
55
20
30
40
50
60
70
80
90

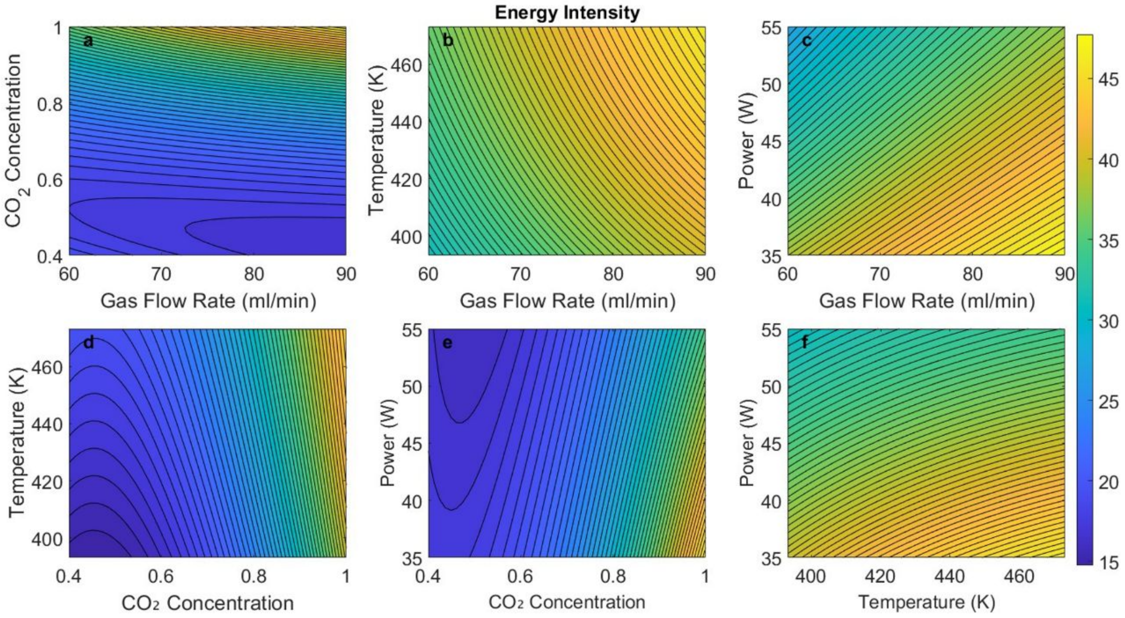
Energy Intensity
a
b
c
d
e
f
CO2 Concentration
Gas Flow Rate (ml/min)
Temperature (K)
Power (W)
0.4
0.6
0.8
1
60
70
80
90
400
420
440
460
35
40
45
50
55
15
20
25
30
35
40
45

Figure SI2 Contour plots of different metric about reaction performance w/ cat: for varying (a) CO_2 concentration and gas flow rate (b) temperature and gas flow rate (c) plasma power and gas flowrate (d) temperature and CO_2 concentration (e) plasma power and CO_2 concentration (d) plasma power and temperature. All other parameters are set to their nominal values according to the central point of Box Behnken design: 428.15 K for temperature, 97% for CO2 concentration, 77.5 ml/min for total flow rate, and 42.5 W for plasma power.

2.2 The best energy efficiency

In order to find out the best energy efficiency, a brute force optimization that runs a nested loop which basically follow such a procedure:

(1) Divide 4 variables (flowrate, the percentage of CO_2, temperature and power) into same into 50 data point evenly over an interval (The total amount of combination in different conditions is 50^4).

(2) Calculate the energy efficiency in each condition and compare them to find out the best one together with the corresponding condition.

(3) The details code about contour plots and finding the best energy efficiency can be seen in the attached file prediction.m

Table SI 7. Best electrical energy efficiency with different $E_{a,surf}$ and related reaction condition

$E_{a,surf}$ (kJ/mol)	Energy efficiency(%)	Flowrate (ml/min)	CO_2 concentration (%)	Temperature (K)	Plasma power (W)
5	25.03	62.44	1.00	473.15	35.00
6	22.66	60.00	1.00	473.15	35.00
7	20.19	60.00	1.00	473.15	35.00
8	17.77	60.00	1.00	473.15	35.00
9	15.50	60.00	1.00	473.15	35.00
10	13.87	60.00	1.00	473.15	55.00
11	13.36	90.00	0.44	473.15	35.00
12	13.14	90.00	0.43	473.15	35.00
13	12.97	90.00	0.42	473.15	35.00
14	12.85	90.00	0.42	473.15	35.00
15	12.76	90.00	0.42	473.15	35.00
16	12.69	90.00	0.41	473.15	35.00

Table SI 8. Best total energy efficiency with different $E_{a,surf}$ and related reaction condition

$E_{a,surf}$ (kJ/mol)	Energy efficiency(%)	Flowrate (ml/min)	CO_2 concentration (%)	Temperature (K)	Plasma power (W)
5	4.30	70.40	1.00	393.15	55.00
6	3.93	63.06	1.00	393.15	55.00
7	3.59	60.00	1.00	393.15	55.00
8	3.22	60.00	1.00	393.15	55.00
9	2.83	60.00	1.00	393.15	55.00
10	2.46	60.00	1.00	393.15	55.00
11	2.22	60.00	0.69	393.15	55.00
12	2.14	90.00	0.47	393.15	55.00
13	2.11	90.00	0.46	393.15	55.00
14	2.10	90.00	0.46	393.15	55.00
15	2.08	90.00	0.44	393.15	55.00
16	2.08	90.00	0.44	393.15	55.00
